# Coupling the H295R with ERα and AR U2OS CALUX assays enables simultaneous testing for estrogenic, anti-androgenic and steroidogenic modalities

**DOI:** 10.1093/toxsci/kfad052

**Published:** 2023-06-01

**Authors:** Martha S Nikopaschou, Alexandre Félix, Julie Mollergues, Gabriele Scholz, Benoit Schilter, Maricel Marin-Kuan, Karma C Fussell

**Affiliations:** Food Safety Research, Nestlé Research, Lausanne, Switzerland; Food Safety Research, Nestlé Research, Lausanne, Switzerland; Food Safety Research, Nestlé Research, Lausanne, Switzerland; Food Safety Research, Nestlé Research, Lausanne, Switzerland; Food Safety Research, Nestlé Research, Lausanne, Switzerland; Food Safety Research, Nestlé Research, Lausanne, Switzerland; Food Safety Research, Nestlé Research, Lausanne, Switzerland

**Keywords:** steroidogenesis, H295R, CALUX, endocrine activity, endocrine disruption

## Abstract

Endocrine active substances, including steroidogenesis modulators, have received increased attention. The *in vitro* H295R steroidogenesis assay (OECD TG 456) is commonly used to test for this modality. However, current detection methods often fail to capture alterations to estrogen biosynthesis. The present study explored the potential of ERα and AR CALUX bioassays to serve as a detection system for the original H295R assay, as they can quantify lower hormone concentrations and can simultaneously provide information about estrogen- and androgen-receptor activities. Using substances from the original OECD validation study, we obtained lowest observed effect concentrations for steroidogenesis mostly equivalent to those previously reported and sometimes lower for estrogen biosynthesis. However, categorization of many of these substances as receptor (ant)agonists or disruptors of steroidogenesis was difficult because often substances had both modalities, including some where the receptor-mediated activities were identified at concentrations below those triggering steroidogenic effects. When the leading activity was not accounted for, H295R-CALUX assay sensitivity in comparison to the OECD validation study was 0.50 for androgen and 0.78 for estrogen biosynthesis. However, upon reinterpretation of the combined assay results to identify endocrine activities without regard to the modality or direction of effects, assay sensitivity was equal to 1.00. These proof-of-concept study findings indicate the high relevance of this assay for the identification of endocrine active substances with additional valuable mode-of-action information and the capacity to detect smaller changes in estrogen biosynthesis, suggesting that the coupled H295R-CALUX assay has promise for the analysis of samples in a decision-making context.

Endocrine active substances have been receiving increased regulatory and public attention, with current regulatory focus on estrogenic, androgenic, thyroid, and steroidogenic (EATS) modalities (European Chemicals Agency [ECHA] and European Food Safety Authority [EFSA], 2018). Steroidogenesis is the well-characterized process during which steroid hormones are synthesized mainly in the gonads and adrenal glands by a combination of pathways across multiple cell types and tissues *in vivo* (Miller and Auchus, 2011; OECD, 2011; UniProt Consortium, 2021). These hormones can be divided into different classes (progestogens, androgens, estrogens, glucocorticoids, and mineralocorticoids) that control multiple physiological processes, including the development of sexual characteristics (Selvaraj *et al.*, 2018). A schematic representation of steroidogenesis is presented in Figure 1.

**Figure 1.**
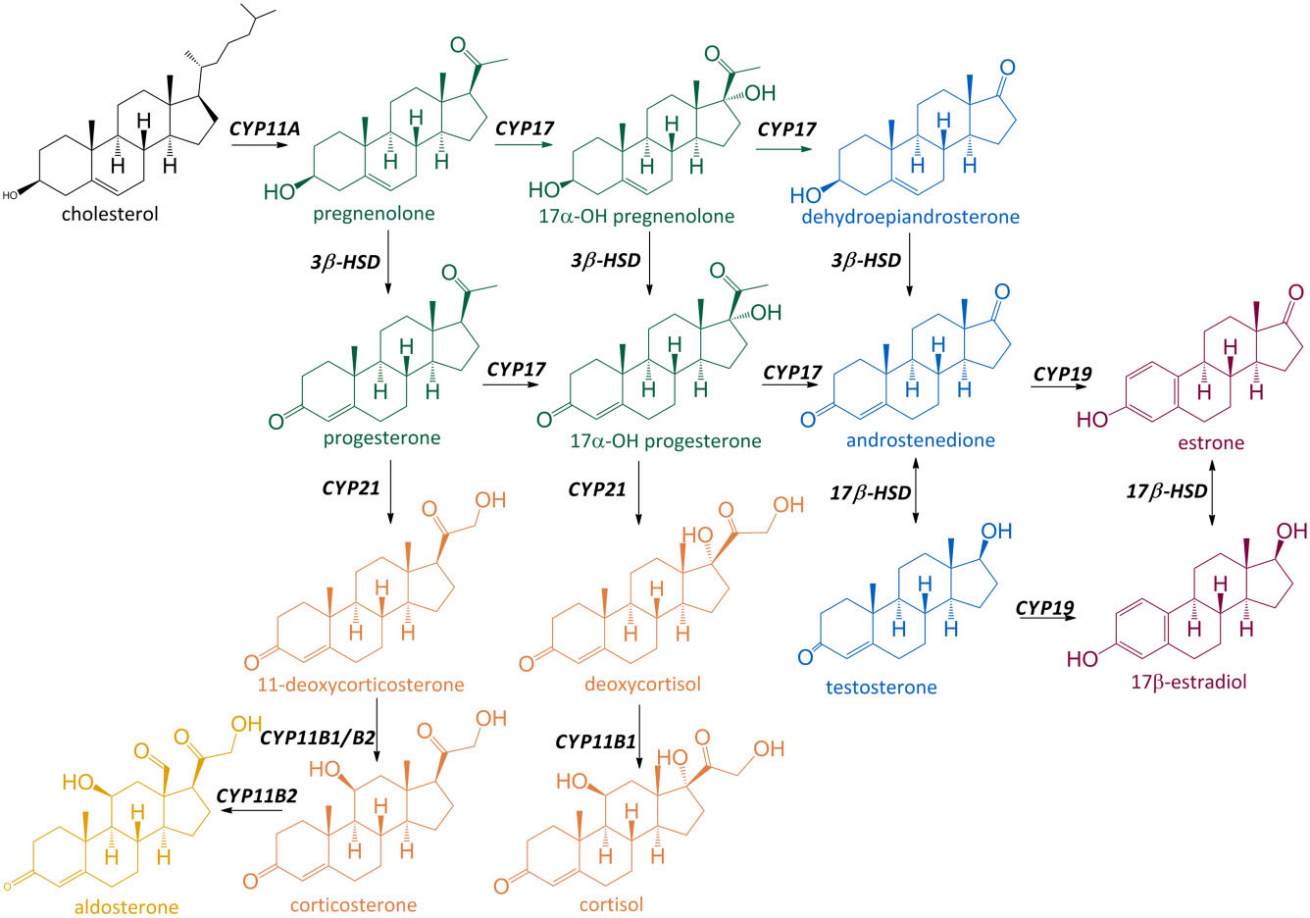
**Pathway of steroidogenesis**. Enzymes catalyzing each reaction are shown in bold. The 5 classes of hormones, namely prostagens, androgens, estrogens, glucocorticoids, and mineralocorticoids are shown in green (pregnenolone, 17α-OH pregnenolone, progesterone, 17α-OH progesterone), blue (dehydroepiandrosterone, androstenedione, testosterone), purple (estrone, 17β-estradiol), orange (11-deoxycorticosterone, deoxycortisol, corticosterone, cortisol), and yellow (aldosterone), respectively.

To model this physiology, the H295R Steroidogenesis *in vitro* assay was developed and validated by the OECD (Organization for Economic Cooperation and Development) as the only widely used protocol to screen for changes in the steroid hormone biosynthesis (OECD, 2010, 2011, 2018; US EPA, 2009). The assay uses a human adenocarcinoma cell line (NCI-H295R) that has the characteristics of zonally undifferentiated human fetal adrenal cells, possesses all the enzymes involved in the steroidogenic pathway, and secretes steroid hormone end products into the surrounding cell culture medium; including the sex steroids 17β-estradiol (E2) and testosterone (T) (Gazdar *et al.*, 1990). Any interference with sex steroid production is detected as a change in these hormone levels, which can be quantified in the cell culture supernatant in various ways (OECD, 2011; US EPA, 2009). Enzyme-Linked Immuno-Sorbent Assay (ELISA), Radio Immunoassay (RIA), and Liquid Chromatography-Mass Spectrometry (LC-MS) were used in the original OECD validation study of the assay (Hecker *et al.*, 2011); other methods such as Gas Chromatography-Mass Spectrometry (GC-MS) are also reported (Nakano *et al.*, 2016; Nielsen *et al.*, 2012; Rijk *et al.*, 2012). Limits of quantification equal to 50 and 8 pg/ml for T and E2, respectively, are recorded for immunoassays (Hecker *et al.*, 2006, 2007; Higley *et al.*, 2010); for mass spectrometric methods the range is wider, between 10 and 1126 pg/ml for T and 3–1000 pg/ml for E2 (Coady *et al.*, 2014; Kang *et al.*, 2013; Karmaus *et al.*, 2016; Nielsen *et al.*, 2012; Repouskou *et al.*, 2019; Strajhar *et al.*, 2017). The limits of quantification of all these methods only marginally meet the OECD method sensitivity criteria (100 pg/ml for T and 10 pg/ml for E2) (OECD, 2011), which were set to ensure the detection of both decreases, as well as increases from the basal hormone production. During the OECD validation trial, basal T production between 1409 and 7229 pg/ml was reported using these methods (2010), enabling the consistent detection of both decreases and increases in T production. On the contrary, basal E2 production by H295R cells lies between 13 and 311 pg/ml, very close to the detection limits of the above-mentioned methods (OECD, 2010). This can be problematic for “quantifying the decreased production of this hormone with regard to the identification of weak inhibitors” (Hecker *et al.*, 2011).

The estrogen receptor alpha (ERα) and androgen receptor (AR) U2OS Chemical Activated LUciferase gene eXpression (CALUX) bioassays could be a promising alternative to the above methods, as limits of quantification equal to 0.7 pg E2/ml and 4.1 pg dihydrotestosterone/ml (equivalent to 24.1 pg T/ml; Sonneveld *et al.*, 2006) have been reported (Pannekens *et al.*, 2019). Moreover, the increased responsiveness of the bioassays to the biological effects of substances present at low concentrations means that slight changes in hormone levels can be readily discerned (Fussell *et al.*, 2021; Leusch *et al.*, 2017; Wang *et al.*, 2014a). In addition, combining the CALUX and the H295R assays can provide information about direct estrogenic and/or anti-androgenic activity, without additional testing. On a practical note, the high similarity in the composition of the culture media for the CALUX and the H295R cells suggests that CALUX may be a compatible detection method for the H295R steroidogenesis test, signifying an opportunity to simultaneously test for multiple endocrine modalities.

The present study assessed ERα and AR CALUX bioassays as an alternative detection system for the original H295R assay. H295R cells were initially exposed to the core chemicals tested during the OECD validation, some of which also directly affect the estrogen and/or androgen receptor. Subsequently CALUX cells were exposed to the cell culture medium-supernatant from the H295R assay to detect the induction or inhibition of estrogen and androgen production by measuring reporter-gene luminescence. At the same time, ERα and AR CALUX cells were directly exposed to the abovementioned chemicals in the same plate to account for direct effects on the receptor.

## Materials and methods

###  

#### Chemicals

17β-estradiol (E2) (CAS No. 50-28-2), aminoglutethimide (CAS No. 125-84-8), atrazine (CAS No. 1912-24-9), benomyl (CAS No. 17804-35-2), bisphenol A (BPA) (CAS No. 80-06-7), butylparaben (CAS No. 94-26-8), cadmium chloride (CAS No. 10108-64-2), dihydrotestosterone (DHT) (CAS No. 521-18-6), dimethylsulfoxide (DMSO) (CAS No. 67-68-5), Dulbecco’s Phosphate Buffered Saline (PBS), flutamide (FLT) (CAS No. 13311-84-7), forskolin (CAS No. 66575-29-9), human chorionic gonadotropin (HCG) (CAS No. 9002-61-3), letrozole (CAS No. 112809-51-5), menadione (CAS No. 58-27-5), molinate (CAS No. 2212-67-1), perfluorooctanesulfonic acid (PFOS) (CAS No. 2795-39-3), prochloraz (CAS No. 67747-09-5), and trilostane (CAS No. 13647-35-3) were all purchased from Sigma-Aldrich (Buchs, Switzerland). (For more information see Supplementary Table 1).

#### Cell culture

The human adrenocortical carcinoma cell line NCI-H295R [H295R] was obtained from the American Type Culture Collection (ATCC CRL-2128; ATCC, Manassas, Virginia). H295R cells were cultured in 75 cm^2^ culture flasks for 4–5 passages prior to cell-bank cryopreservation (2×10^6^ cells/cryovial). As needed, thawed cells were cultured at 37°C with 5% CO_2_ and 100% humidity in Dulbecco’s Modified Eagle’s Medium with Ham’s F-12 Nutrient mixture with phenol red (DMEM/F12, Gibco, Bleiswijk, The Netherlands) supplemented with 2.5% v/v Nu-Serum (Corning, Tewksbury, Massachusetts) and 1% v/v solution of penicillin-streptomycin (100 U/ml penicillin and 0.1 mg/ml streptomycin) (PAN Biotech, Aidenbach, Germany), plus either 1% v/v ITS+ Premix (Corning, Tewksbury, Massachusetts) or its equivalent: 1% v/v ITS Premix (also Corning), 0.00535 mg/ml linoleic acid (Sigma-Aldrich, Buchs, Switzerland), and 1.25 mg/ml bovine serum albumin (Sigma-Aldrich, Buchs, Switzerland). The H295R cells were subcultured every 3–4 days, when the confluency was approximately 80% (dilution ratio 1:2–1:4). After 3 passages, a quality control plate was seeded for the evaluation of the cells’ performance.

The ERα and (anti-)AR CALUX U2OS cells, obtained under license from BioDetection Systems (Amsterdam, The Netherlands), were cultured at 37°C with 5% CO_2_ and 100% humidity in 75 cm^2^ culture flasks using sterile-filtered Dulbecco’s Modified Eagle’s Medium with Ham’s F-12 Nutrient mixture with phenol red (DMEM/F12, Gibco, Bleiswijk, The Netherlands), 7.5% v/v Fetal Bovine Serum (Gibco, Paisley, UK), 0.4% v/v G418 Solution (0.2 mg/ml geneticin) (Roche Diagnostics, Mannheim, Germany), 1% v/v nonessential amino acids (Gibco, Paisley, UK), and 1% v/v solution of penicillin-streptomycin (100 U/ml penicillin and 0.1 mg/ml streptomycin) (PAN Biotech, Aidenbach, Germany) (OECD, 2016). As with H295R cells, the cells were passed every 2–4 days, when the confluency was about 80% (dilution ratio 1:2–1:10).

#### Bioassay procedure

##### H295R seeding and treatment

**Figure 2.**
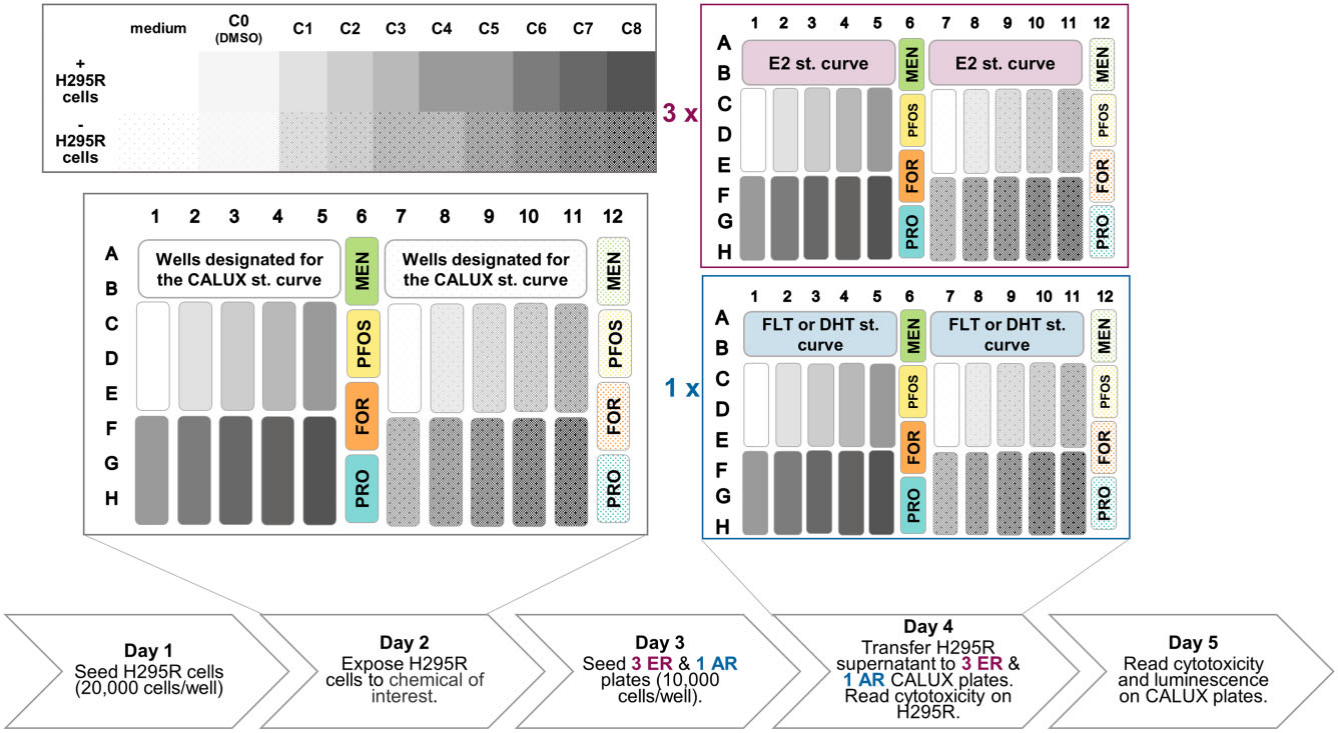
**Schematic representation of the experimental procedure**. Briefly, on day 1, columns 1–5 from row C to row H and column 6 from row A to row H were seeded with H295R cells. The rest of the plate was supplemented only with medium to check whether the chemicals will exert (ant)agonistic activity in the CALUX cells. On day 2, both H295R cells and sham medium wells were treated with test substance and control treatments. On day 3, 1 AR CALUX and 3 ER CALUX plates were seeded, in order to be able to test multiple dilutions in the ER assay. On day 4, the supernatant from the H295R plate was diluted into the CALUX plates and the viability of the H295R cells was assessed. Lastly on day 5, the viability of the CALUX cells was assessed in all 4 plates, which were then washed, lysed and the induction of the luciferase gene was evaluated as luciferin luminescence.

H295R cells at passages 3–4 (for quality control plates) or passages 4–11 after thawing (for test plates) were subcultured as indicated above, resuspended in H295R assay medium composed of Dulbecco’s Modified Eagle’s Medium with Ham’s F-12 Nutrient mixture without phenol red (Gibco, Bleiswijk, The Netherlands), 5% Charcoal stripped serum (Gibco, New York), 1% v/v ITS+ Premix—or its equivalent, as mentioned above—and 1% v/v solution of penicillin-streptomycin. Cells were counted using a Cellometer automatic cell counter (Nexcelom, Lawrence, Massachusetts) and seeded at the recommended density of 20 000 cells/well in 100 μl total volume (OECD, 2011), in the left half (columns 1–6) of a 96-well white clear-bottom plate (Corning, Kennebunk, Maine), except for the wells designated for the CALUX standard curve (rows A and B, columns 1–5 and 7–11 in the H295R plate of Figure 2). These wells, as well as those on the right half of the plate (columns 7–12) were then filled with H295R assay medium without cells. Plates were cultured for 24 h at 37°C with 5% CO_2_ and 100% humidity.

After this acclimatization period, the assay medium was aspirated and replaced with new assay medium containing test compound in DMSO (0.1% DMSO final), except for the lyophilized, buffered solution of human chorionic gonadotropin which was initially reconstituted using sterile milli-Q water and further diluted using PBS. For the quality control plates, forskolin (0.01, 0.1, and 1 μΜ), PFOS (10, 30, and 100 μΜ), and prochloraz (0.001, 0.1, and 1 μΜ final) were used as positive control substances for steroidogenesis; menadione (50 and 10 μΜ final) was also incorporated as a positive control for cytotoxicity and vehicle- and medium-only negative controls were included as well. In the test plates, 8 sample concentrations were assayed in technical triplicates, together with triplicate vehicle (C0) and medium-only controls and duplicate forskolin (1 μΜ), PFOS (100 μΜ), prochloraz (1 μM), and menadione (10 μΜ). The tested substances were chosen from the core chemicals of the OECD validation study with some modifications: (1) nonoxynol-9 was excluded from the dataset due to uncertainties with regards to the exact structure of the oligomer (Haggard *et al.*, 2018), (2) bisphenol A (BPA) was added as it is considered one of the proficiency chemicals in the OECD Test Guideline 456 (OECD, 2011), and (3) ethylene dimethanesulfonate was replaced by cadmium chloride as an alternative cytotoxic chemical. Each concentration was tested on the left side of the plate to determine the H295R steroidogenic response, as well as in the sham wells on the right side of the plate to control for direct activity of the test chemical at the nuclear receptors. A summary of the experimental procedure together with the distribution of the chemicals within the plates are presented in Figure 2.

##### U2OS CALUX quantification of alterations to estrogen and androgen biosynthesis

After 24 of the 48 h H295R cell exposure, the U2OS CALUX cells were subcultured; resuspended in CALUX assay medium composed of Dulbecco’s Modified Eagle’s Medium with Ham’s F-12 Nutrient mixture without phenol red, 5% charcoal-stripped serum, 1% nonessential amino acids, and 1% solution of penicillin-streptomycin; counted and seeded at a density of 10 000 cells/well in all wells of a clear-bottom white 96-well plate (medium volume per well = 100 μl). For each treated H295R plate, 3 ERα CALUX plates (passages 12–42) and 1 AR CALUX plate (passages 15–41) were seeded, to test, respectively, 3 and 1 dilutions of H295R treatment.

After 24 h of acclimatization at 37°C with 5% CO_2_ and 100% humidity, the CALUX wells, except for those to be exposed to the CALUX standard curves, were treated with diluted H295R-plate supernatant (H295R medium with treatment substance, DMSO, and any steroid hormones produced by the H295R cells) on top of the already existing 100 μl of CALUX assay medium. The remaining wells were exposed to the appropriate CALUX standard curve (described below). The levels of hormones produced during steroidogenesis were then quantified as reporter gene activities; these can be directly compared with the activities of the supernatant of the sham wells lacking H295R cells to separate the effects of steroidogenesis from any treatment-related direct activities at the receptor. In addition to the technical triplicates on each plate, each test substance was assessed 3 different times as independent biological replicates.

In the case of ERα, the most likely direct action is receptor agonism (though antagonists do occur), so the sham wells were tested in the ERα CALUX in agonist mode, using 17β-estradiol (E2) as the CALUX standard curve (OECD, 2016), diluted in a mixture of 1:1 CALUX assay and H295R assay medium in a final concentration range between 0.5 and 500 pM. After aspiration of the pre-existing medium, these were used to treat in duplicate the wells reserved for the standard curve on all 3 ERα plates. The remaining wells of each plate then received 1 of 3 combinations of H295R supernatant and H295R assay medium containing DMSO, added directly to the well without aspiration: 30 μl of the H295R supernatant with 70 μl of H295R assay medium, 28 μl supernatant with 72 μl medium, and 25 μl supernatant with 75 μl medium, unless otherwise stated (final dilution of H295R supernatant on ER CALUX cells: 6.6- to 8-fold in 0.05% DMSO).

In the case of AR, receptor agonists are rare (except for pharmaceuticals), so test substances were generally assessed against an AR-antagonism CALUX assay in the sham wells. Flutamide (FLT) was used as the CALUX standard (OECD, 2020). FLT solutions of a series of 0.005–50 μΜ final concentration were made in a mixture of 1:1 CALUX assay medium: H295R assay medium, supplemented with 0.3 nM dihydrotestosterone (DHT), equivalent to the DHT EC_50_. These were used to treat, in duplicate, the standard curve wells of the AR CALUX plate after aspiration of the pre-existing medium. For the test wells with H295R cell exposure (agonist mode), 67 μl of the H295R supernatant and 33 μl of H295R assay medium containing DMSO were added to the 100 μl of CALUX assay medium already in the well (0.1% DMSO final). This 3-fold dilution was chosen such that the basal hormone production by the H295R cells approximates a 50% response in the AR CALUX assay (based on historical data). For the sham test wells (antagonist mode), a 1.8 nM DHT agonist solution in H295R assay medium was prepared and 33 μl of this solution were added together with 67 μl of the H295R supernatant on top of the 100 μl of CALUX assay medium (0.1% DMSO).

However, in some experiments, androgen production should be quantitated, eg, quality control experiments, whereas in others, the possibility of direct androgenic activity should be excluded in order to confirm that the increase in the AR CALUX response is due to increased androgen production in H295R cells. In these experiments, the AR CALUX was performed entirely in agonist mode (no agonist co-treatment) and the standard curve contained varying concentrations of DHT. In a protocol similar to that for ERα, stock solutions of DHT in DMSO were diluted in a mixture of 1:1 CALUX assay and H295R assay medium to a final concentration range of 5 pM–0.5 μΜ. After aspiration of the pre-existing medium, these were used to treat in duplicate the wells reserved for the standard curve on the AR plate. For the test wells, 67 μl of the H295R supernatant and 33 μl of H295R assay medium containing DMSO were added to the 100 μl of CALUX assay medium (3-fold dilution of H295R supernatant, 0.05% DMSO).

##### Quantification of H295R and U2OS cell viability

After the treatment of the CALUX cells, cytotoxicity was assessed in the original H295R plate using the RealTime-Glo MT Cell Viability assay (Promega, Dübendorf, Switzerland) as previously described (Fussell *et al.*, 2021; Marin-Kuan *et al.*, 2017). Briefly, 100 μl of NanoLuc enzyme and MT Cell Viability Substrate diluted 16 000-fold were added to the remaining 50 μl in each well. After 10 min at 37°C, luminescence was measured in a CentroXS LB960 (Berthold Technologies, Zug, Switzerland). A day after exposure of the CALUX cells to H295R supernatant (24 ± 2 h), 150 μl of supernatant were discarded from the CALUX plates and the cell viability measurement was repeated for the CALUX cells.

##### Measurement of estrogen and androgen receptor activity

Following the cytotoxicity measurement, each CALUX plate was washed 4 times with 300 μl PBS in a 405 LS microplate washer (BioTek Instruments, Winooski, Vermont) to completely remove the RealTime-Glo solution. The residual PBS was manually removed from the wells and the cells were lysed for at least 5 min on a plate shaker after the addition of 30 μl lysis buffer (BioDetection Systems, Amsterdam, The Netherlands) (1% Triton X-100, 10% glycerol, 25 mM Tris buffer pH 7.8, 2 mM DTT, and 2 mM CDTA). Luminescence was measured well-by-well in the CentroXS LB960, 4 s after the automatic addition of 100 μl of BDS illuminate-mix (20 mM tricine buffer pH 7.8, 1.07 mM (MgCO_3_)_4_Mg(OH)_2_, 10 μM EDTA, 1.5 mM DTT, 539 μM D-luciferin, and 5.49 mM ATP) (BioDetection System, Amsterdam, The Netherlands).

#### Data processing

##### Cell viability

Cell viability, recorded as relative light units (RLUs), was used to calculate % cell viability in Microsoft Excel 365 by normalizing each measurement to the mean RLU of the vehicle control wells. For the H295R plate, this corresponds to the wells containing H295R cells exposed to 0.1% DMSO, but on the CALUX plates this translates to the wells of the E2, DHT, and FLT standard curves containing only the DMSO vehicle. Subsequently, the mean % viability for each tested concentration was calculated from the 3 technical replicates in each biological experiment. These mean viabilities from the 3 biological replicates were used to derive the mean of means, with its respective Standard Error of Means (SEM). Concentrations with less than 80% cell viability, on average, were considered cytotoxic; activities with concomitant cytotoxicity were not necessarily dropped, but the cytotoxicity was taken into consideration during results interpretation.

##### CALUX reporter gene activities

To calculate % H295R estrogen and androgen production, the background ERα and AR reporter gene activity, computed as the mean RLU of the vehicle controls in the CALUX standard curve, was subtracted from the raw RLU value reported for each well. Subsequently, these values were normalized to either the maximum mean RLU for each concentration of the standard curve (largest E2 or DHT response in agonist mode) or the agonist-only well of the FLT concentration-response curve (for AR antagonist mode). These values, expressed as % relative induction (%RI), were then re-normalized to the estrogen or androgen activity of H295R cells treated only with DMSO (% of basal hormone production). Values above 100% represent induction in the respective hormone synthesis; values below indicate inhibition of these pathways. The same methodology was also used to normalize values for direct receptor activity. In cases of direct receptor agonism, statistically significant escalations from 0% represent induction, whereas reductions from 100% indicate antagonistic effects in those assays.

To determine the statistical relevance of any steroidogenic changes, the 3 technical replicates across all 3 biological experiments were pooled into a single dataset for each test chemical (*n* = 9). These pooled data were evaluated for normality using a Shapiro-Wilk test, Q-Q plots, and histograms (Supplementary Figure 1), both prior to and after a log transformation. Due to the absence of normality in most of the data and to evaluate all the chemicals based on the same methodology, statistical significance of the pooled data was evaluated nonparametrically. In accordance with OECD recommendations, a Kruskal-Wallis test was applied, followed by a series of Mann-Whitney U pairwise comparisons in R (version 4.04; the script is available in the Supplementary Material) (Hecker *et al.,* 2011). In the first pairwise comparison, the wells containing H295R treated cells were compared with the basal steroidogenesis by the H295R cells (DMSO control, set to 100%) to determine which alterations in steroidogenesis were statistically significant (*.01<*p*≤ .05, **.001<*p* ≤ .01, ***.0001 <* p*  ≤ .001, ****p ≤ .0001).

For direct receptor activities a second comparison was performed, in which all sham groups (wells containing supernatant from wells lacking H295R cells) were compared with the sham control (well with supernatant from DMSO control lacking H295R cells) containing either vehicle (agonist mode) or 0.3 nM DHT (antagonist mode) (#.01<*p* ≤ .05, ##.001<*p* ≤ .01, ###.0001<*p* ≤ .001, ####*p* ≤ .0001).

Chemicals were characterized for steroidogenic effects with reference to the OECD guidelines (2011). Concentrations were classed as positive only if 2 consecutive concentrations and/or the maximum noncytotoxic concentration was statistically significantly different from the control, but only if (ant)agonistic receptor activity was also statistically and biologically insignificant at the same nominal concentration on the CALUX cells. The Lowest Effect Concentration (LOEC) was defined as the lowest test concentration to be statistically significantly different from the control; in the H295R portion of the assay, these concentrations are reported as the final treatment concentrations in the H295R plate, whereas for the receptor-mediated effects measured in the sham wells, the additional dilution into the CALUX plate was also considered. Due to the need for different dilution factors in ERα CALUX across the various experiments in each biological triplicate for a respective chemical, an average dilution factor was applied for each substance concentration. This practical approach introduces up to 23% error in the reported concentrations, a level well-within the inherent variation of the CALUX assay.

To plot the data, the technical replicates within each of the 3 biological experiments were averaged and transferred to GraphPad Prism (version 9.1.2, San Diego, CA) for visualization. Graphs with closed symbols depict the mean of means for % H295R estrogen/androgen synthesis as a function of treatment and concentration, whereas graphs with open symbols are used to depict measurements of cell viability. Statistical significance is reported as described above.

#### Comparisons between the combined H295R-CALUX and existing *in vitro* and *in vivo* data

Ten of the chemicals used in this study (aminoglutethimide, atrazine, benomyl, butylparaben, forskolin, HCG, letrozole, molinate, prochloraz, and trilostane), belong to the core chemicals of the OECD validation program. BPA was added to this dataset, as it is also considered a reference chemical by the OECD (OECD, 2011). H295R-CALUX data for these 11 substances were compared with the findings observed in the OECD H295R validation study (Hecker *et al.*, 2011). A further 2 compounds, cadmium chloride and PFOS, were not analyzed by the OECD and therefore initial comparisons were not possible (Figure 6; Tables 4 and 5). However, these substances were included in broader literature comparisons for endocrine activities and cytotoxicity (Tables 6 and 7). In addition*, in vivo* data comparisons were performed for 10 of the 11 OECD referenced chemicals (aminoglutethimide, atrazine, benomyl, BPA, butylparaben, HCG, letrozole, molinate, prochloraz, and trilostane), as no *in vivo* data are available for forskolin. These *in vivo* data were retrieved from Table 5 of the multi-laboratory validation study (Hecker *et al.*, 2011) and are also referenced in Tables 6 and 7 of this study.

**Table 4.** Sensitivity, specificity, and accuracy of the H295R-CALUX when compared with the OECD study and with known endocrine activities *in vitro*

	H295R-CALUX versus OECD Validation			H295R-CALUX versus Known Endocrine Activities *In Vitro*		
Hormone	Sensitivity	Specificity	Accuracy	Sensitivity	Specificity	Accuracy
Androgens/T	0.50	0.71	0.64	1.00		0.91
Estrogens/E2	0.78		0.82	1.00		1.00

**Table 5.** Sensitivity, specificity, and accuracy of the H295R-CALUX and of the OECD *in vitro* study when compared with *in vivo* data

	H295R-CALUX versus *In Vivo* Data			*In Vitro* OECD Validation versus *In Vivo* Data		
Hormone	Sensitivity	Specificity	Accuracy	Sensitivity	Specificity	Accuracy
Androgens/T		0.86	0.80		0.55	0.60
Estrogens/E2	1.00	0.80	0.90	1.00	0.33	0.60

**Table 6.** Comparison of H295R androgen biosynthesis data obtained in this study with data from the OECD validation program and *in vivo* data referenced in the OECD validation study.

Chemical	Testosterone/androgen activity					Direct anti-androgenicity	
	Effect			LOEC (μΜ)		LOEC (μΜ)	
	This study	H295R validation study (Hecker et al., 2011)	In vivo data^a^	This study (μΜ in H295R Wells)	H295R validation study (Hecker et al., 2018)	This study (μΜ final in the CALUX Wells)	Literature (U2OS CALUX)
Prochloraz	Down	Down	Down*^b^*	0.03*^c^*	0.0001–0.1	nd^*d*^	≥0.3*^e^*
Forskolin	Down	Up	—	3	1–10	nd	—
PFOS	Up	—*^f^*	Up/down*^g^*	100	—	nd	—
Aminoglutethimide	Down	Down	Up*^h^*	100	10–100	nd	—
Trilostane	Down	Up	Down*^i^*	0.003	0.01–1	nd	—
HCG	nd	nd	nd*^j^*	nd	nd	nd	—
Atrazine	nd*^k^*	nd/up*^l^*	nd*^m^*	(100)	nd/1	100	Weak positive*^n^*
Letrozole	nd*^k^*	Down	Up*^o^*	(30)	100	33	—
Molinate	nd*^k^*	nd*^p^*	nd*^q^^,^^r^*	(100)	nd/100	67	Positive*^s^*
BPA	nd*^k^*	Down	nd*^t^*	(3)	10	0.3	≥0.3*^n^*
Benomyl	nd*^k^*	nd	nd*^u^*	nd	nd	10	Positive*^s^*
Butylparaben	nd*^k^*	nd	nd*^v^*	(10)	nd	10	≥3*^n^*
Cadmium Chloride	nd	—^w^	Down*^x^*	nd	—	nd	nd*^y^*

nd, no effect could be detected; —, no data available. Data in parentheses refer to effects due to direct interaction with the receptor.

a The *in vivo* data are obtained from the references cited at Table 5 of the validation study (Hecker *et al.*, 2011), supplemented with an additional study for PFOS and cadmium chloride.

b Brande-Lavridsen *et al.* (2008) and Vinggaard *et al.* (2005).

c Smallest concentration tested.

d up to 1.3 μΜ (highest concentration tested).

e van der Burg *et al.* (2010): In this study, dose-response curves and IC_50_ values are reported for some chemicals without statistical information. ≥ is used to indicate the point where a difference from the control starts being visible in the dose-response curve. The average IC_50_ value measured for prochloraz is 1.76 μΜ.

f This chemical was not tested in the validation study of Hecker *et al.* (2011). Data from the literature suggest an increase in T levels (Kraugerud *et al.*, 2011; Pinto *et al.*, 2018; van den Dungen *et al.*, 2015).

g Alam *et al.* (2021), Li *et al.* (2018), and Zhao *et al.* (2014).

h Berman and Laskey (1993) and Monteiro *et al.* (2000).

i Jungmann *et al.* (1983).

j Characterized as no effect downstream of the gonadotropin hormone receptors (follicle-stimulating hormone receptor and luteinizing hormone receptor) because only effects downstream of the gonadotropin hormone receptors (follicle-stimulating- and luteinizing hormones) are relevant for the H295R assay (Hecker *et al.*, 2011).

k Chemical resulted in concentration dependent decrease in both steroidogenic and direct-receptor assay. Therefore, it was characterized as negative for steroidogenesis in the H295R-CALUX due to the presence of direct receptor activity.

l Significant increase in 1 laboratory and nonsignificant increase in 2 other laboratories at 100 μM.

m Spanò *et al.* (2004) and Wetzel *et al.* (1994).

n Wang *et al.* (2014b): In this study, dose-response curves and IC_50_ values are reported for some chemicals without statistical information. ≥ is used to indicate the point where a difference from the control starts being visible in the dose-response curve. IC_50_ values for BPA and butylparaben are 1.5 and 7.1 μΜ, respectively. Atrazine is characterized as a weak antiandrogen with no defined IC_50_ value due to poor fitting of the dose-response model to the data.

o Kumru *et al.* (2007).

p Significant decrease in 1 laboratory at the highest concentration (100 μΜ).

q Inconclusive results.

r Ellis *et al.* (1998).

s van der Burg *et al.* (2015): Molinate is reported with an EC_50_ of 1 μΜ and benomyl with an EC_50_ of 10 μΜ. Νο LOECs or dose-response curves are reported.

t Yamasaki *et al.* (2002).

u Carter and Laskey (1982) and Spencer *et al.* (1996).

v Taxvig *et al.* (2008).

w This chemical was not tested in the validation study of Hecker *et al.* (2011). Data from the literature suggest no significant effects on H295R cells on noncytotoxic concentrations (Knazicka *et al.*, 2015).

x Lau *et al.* (1978).

y Marin-Kuan *et al.* (2017).

**Table 7.** Comparison of H295R estrogen biosynthesis data obtained in this study with data from the OECD validation program and *in vivo* data referenced in the OECD validation study

Chemical	Estradiol/estrogen					Direct estrogenicity	
	Effect			LOEC (μΜ)		LOEC (μΜ)	
	This study	H295R validation study (Hecker *et al.*, 2011)	*In vivo* data^*a*^	This study (μΜ in H295R Wells)	H295R validation study (Hecker *et al.*, 2018)	This study (μΜ final in the CALUX Wells)	Literature (U2OS CALUX)
Prochloraz	Down	Down	Down*^b^*	0.03	0.1–1	nd	—
Forskolin	Up	Up	—	0.3*^c^*	0.01–0.1	nd	—
PFOS	Up	—*^d^*	Down*^e^*	100	—	nd	—
Aminoglutethimide	Down	Down	Down*^f^*	3	10–100	nd	—
Trilostane	Down and then up	Up	Down/nd*^g^*	0.1	0.1–100	nd	—
HCG	nd	nd	nd*^h^*	nd	Nd	nd	—
Atrazine	Up	Up	Up*^i^*	1	0.1–10	nd	nd*^j^^,^^k^*
Letrozole	Down	Down	Down*^l^*	3×10^−5^	10^−4^–0.01	nd	—
Molinate	Up	Up	nd*^m^*	200	100	nd	nd/positive*^n^*
BPA	nd*^o^*	Up	nd*^p^*	(3)	1–10	0.16	≥0.1*^j^*
Benomyl	nd	nd	nd*^q^*	nd	nd	nd	nd*^n^*
Butylparaben	nd*^o^*	Up*^r^*	nd*^s^*	(30)	10	4.2	Positive*^j^*
Cadmium chloride	nd	—*^t^*	Down*^u^*	nd	—	nd	—

nd, no effect could be detected; —, no data available. Data in parentheses refer to effects due to direct interaction with the receptor.

a The *in vivo* data are obtained from the references cited at Table 5 of the validation study (Hecker *et al.*, 2011), supplemented with additional information for PFOS, trilostane, and cadmium chloride.

b Brande-Lavridsen *et al.* (2008) and Vinggaard *et al.* (2005).

c By random chance, the response at 0.03 μΜ was statistically significant, but the next tested concentration (0.1 μΜ) was not; therefore, we counted 0.3 μΜ as LOEC, because it is this concentration that is followed only by significant hits.

d This chemical was not tested in the validation study of Hecker *et al.* (2011). Data from the literature suggest increase in E2 levels (Kraugerud *et al.*, 2011; Pinto *et al.*, 2018; van den Dungen *et al.*, 2015).

e Feng *et al.* (2015).

f Berman and Laskey (1993) and Monteiro *et al.* (2000).

g Beardwell *et al.* (1985) and Jungmann *et al.* (1983).

h No effect downstream of the gonadotropin hormone receptors (follicle-stimulating hormone receptor and luteinizing hormone receptor) (Hecker *et al.*, 2011).

i Spanò *et al.* (2004) and Wetzel *et al.* (1994).

j Wang *et al.* (2014b): In this study, dose-response curves and EC_50_ values are reported without statistical information. ≥ is used to indicate the point where a difference from the control starts being visible in the dose-response curve. EC_50_ values for BPA and Butylparaben are 0.27 and 2.9 μΜ, respectively.

k Besselink (2015).

l Kumru *et al.* (2007).

m Ellis *et al.* (1998).

n van der Burg *et al.* (2015): For benomyl no effect on ERα CALUX is reported. For molinate the observed estrogenicity has an EC50 of 631 μΜ, well above the 42.5 μΜ highest concentration used in f this study.

o Chemical resulted in concentration dependent increase in both steroidogenic and direct-receptor assay. Therefore, it was characterized as negative for steroidogenesis in the H295R-CALUX due to the presence of direct receptor activity.

p Yamasaki *et al.* (2002).

q Carter and Laskey (1982) and Spencer *et al.* (1996).

r Increase in 2 laboratories.

s Taxvig *et al.* (2008).

t This chemical was not tested in the validation study of Hecker *et al.* (2011). Data from the literature suggest no significant effects on H295R cells on noncytotoxic concentrations (Knazicka *et al.*, 2015).

u Das and Mukherjee (2013).

To summarize assay performance, 3 sets of confusion matrices were created from these data for estrogens and androgens, as performed by Haggard *et al.* (2018) with some modifications. Within each set, only 1 matrix was constructed for each group of hormones, taking full advantage of the available dataset. In the first set of confusion matrices, the results of the present study were compared with the OECD study as the source of “true” positives, with equivocal data from the OECD study considered positive for effects only if the respective effect was detected with significance by at least 2 laboratories. Moreover, the direction of steroidogenic effect also mattered; if it matched (induction with induction or inhibition with inhibition), the result was considered a true-positive, but if the effect direction differed between reported and current data, the finding was considered a false-positive. The second pair of confusion matrices was constructed with the broader aim of identifying endocrine activities without regard to modality. These were similar to the first set, except that previously reported CALUX results were added to the validation study findings, such that the combined estrogenicity, anti-androgenicity, and steroidogenesis data indicated “true” *in vitro* endocrine activity. These outcomes were then compared with the results of the present study without regard to either the modality or the direction of effect. The last pair of confusion matrices were constructed as a parallel to the assessment used to evaluate the predictivity of the H295R assay in the OECD validation trial. In order to compare the capacities of the original and coupled H295R assay to predict *in vivo* effects, the results of the present study were compared with the *in vivo* data summarized in the OECD study; again, the direction of the perturbation was also taken into account when sorting between true- and false-positive results. For each pair of confusion matrices, sensitivity, specificity, and accuracy were calculated as in Haggard *et al.* (2018) according to the following formulas, but only in cases where a minimum of 4 substances are included in the calculation:


$$\mathrm{sensitivity}\;\left(\mathrm{true}\;\mathrm{positive}\;\mathrm{rate}\right)=\frac{\mathrm{true}\;\mathrm{positives}}{\mathrm{true}\;\mathrm{positives}+\mathrm{false}\;\mathrm{negatives}}$$

$$\mathrm{specificity}\;\left(\mathrm{true}\;\mathrm{negative}\;\mathrm{rate}\right)=\;\frac{\mathrm{true}\;\mathrm{negatives}}{\mathrm{true}\;\mathrm{negatives}+\mathrm{false}\;\mathrm{positives}}$$

$$\mathrm{accuracy}=\;\frac{\mathrm{true}\;\mathrm{positives}+\mathrm{true}\;\mathrm{negatives}}{\mathrm{total}\;\mathrm{number}\;\mathrm{of}\;\mathrm{chemicals}\;\mathrm{for}\;\mathrm{effect}\;\mathrm{type}}$$

## Results

**Figure 3.**
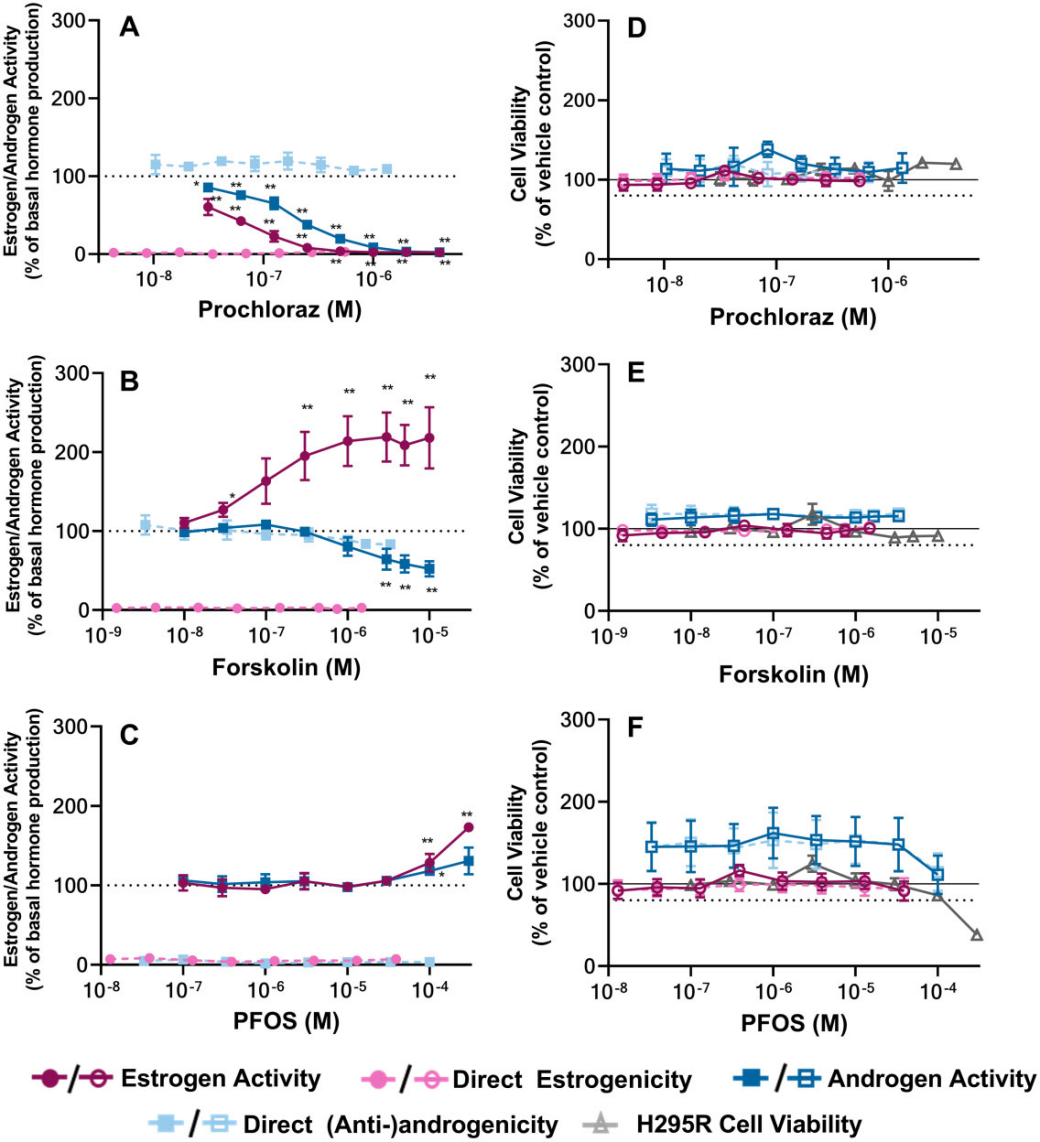
**Effect of Prochloraz, Forskolin, and PFOS on H295R-mediated steroidogenesis and cell viability**. A–C, Apparent induction or inhibition of estrogen and androgen synthesis in the presence (Estrogen/Androgen Activity) and absence (Direct Estrogenicity/(Anti-)androgenicity) of H295R cells in response to treatment with prochloraz (A), forskolin (B), and PFOS (C). Direct receptor-mediated activities were also assessed for these substances in accordance with the overall directionality of effect: ERα agonism (0%=no activity) and AR antagonism (100%=no activity) for forskolin and prochloraz, as well as ERα and AR agonism (0%=no activity) for PFOS. Additional same-well cell viability measurements were also made for the H295R cells, and all ER and AR U2OS CALUX cells (Panels D–F). Reported concentrations reflect either the final well-concentration in the H295R assay (estrogen activity, androgen activity and H295R cell viability) or the mean final concentration in the CALUX wells (direct estrogenicity, direct (anti-)androgenicity and CALUX cell viability). The graphs depict the results of 3 biologically independent experiments made up of 3 technical replicates (*n*=9 when pooled for statistical analysis), with the exception of the direct AR antagonistic effects of prochloraz (and same-well AR CALUX viability measures), where *n*=6. Each point represents the mean of means ± SEM. Significance was evaluated by a Kruskal-Wallis test, followed by a Mann-Whitney *U* test for multiple comparisons. The response at each concentration was compared with basal H295R hormone synthesis when treated with DMSO vehicle (estrogen or androgen activities; *.01<*p*≤.05, **.001<*p*≤.01, ***.0001<*p*≤.001, ****p≤.0001) or the corresponding sham treatment (direct estrogenicity and (anti-)androgenicity; #.01<*p*≤.05, ##.001<*p*≤.01, ###.0001<*p*≤.001, ####*p*≤.0001). Note: For 2/3 forskolin experiments, the treatments were diluted into the ERα CALUX wells using 32 μl of H295R supernatant and 68 μl of DMSO-containing H295R assay medium. For 1/3 PFOS experiments, the treatments were diluted into the ERα CALUX wells using 20 μl of H295R supernatant and 80 μl of DMSO-containing H295R assay medium. These modifications were necessary to achieve hormone levels within the linear range of the CALUX method. The remaining experiments were performed as described in the Materials and Methods section.

The present study coupled the H295R and CALUX bioassays to enhance the detection of alterations to hormone biosynthesis during steroidogenesis testing and provide additional information about potential direct activity on the ERα and AR receptor in a single *in vitro* test. To accommodate the CALUX detection system, methodological refinements were necessary. In particular, the H295R supernatant was diluted 3-fold for AR CALUX and 6- to 8-fold for ERα CALUX to accommodate the more sensitive CALUX system. As recommended by the OECD, prochloraz and forskolin were assessed as positive controls for inhibition and induction of steroidogenesis, respectively. Figure 3 demonstrates the response of AR and ERα CALUX cells after exposure to the supernatant of the treated H295R cells (Estrogen/Androgen activity), as well as the response to the supernatant of the sham wells lacking H295R cells (Direct Estrogenicity/(Anti)androgenicity). Note that in all graphs found in this report, activities resulting from H295R cell treatments are reported as a consequence of the original concentrations used to expose H295R cells, whereas the direct receptor activities and CALUX cell viabilities are reported based on the diluted concentrations used to treat the CALUX cells. For example, the activities resulting from the 1 μΜ forskolin H295R treatment used as a positive control in the combined H295R-CALUX, which were diluted to actual treatment concentrations of 0.33 μΜ in the AR CALUX cells and 0.17 μM in the ERα CALUX cells, are graphed in relation to the original 1 μΜ forskolin H295R treatment for ease of comparison with the H295R viability data. However, the direct receptor activities from the sham wells are graphed in relation to the actual treatment concentration in those wells, such that the leading activity and lowest observed effect concentration can be readily determined. All viabilities are reported in accordance with the actual treatment concentration in the well to make clear any inconsistencies between the viabilities of different cell lines and also to facilitate comparison with the direct receptor-mediated activities. However, it is important to remember that the highest reported concentrations within each panel or pair of panels are related, as are the second highest treatment concentrations and so on.

Prochloraz responded as expected (Figure 3A); a concentration-dependent decrease in both estrogen and androgen levels was observed with H295R treatment. In the case of forskolin (Figure 3B), induction was confirmed only for estrogen production (lowest observed effective concentration, LOEC=0.3 μΜ). Instead, a concentration-dependent reduction in the apparent H295R androgen production was observed, accompanied by small, statistically insignificant AR antagonism after exposure to the supernatant of the sham wells at the same concentrations. This was likely due to a nonspecific effect of forskolin on the CALUX cells.

Consequently, alternative positive controls for induction of both estrogen- and androgen-production by H295R cells were investigated (data not shown). Perfluorooctanesulfonic acid (PFOS) was the only chemical exhibiting an increasing trend for both endpoints, while not directly agonizing AR and ERα. Figure 3C shows statistically significant, but weak increases for both estrogens and androgens after treatment with 100 μΜ PFOS; a more significant increase at 300 μΜ was also observed for estrogens only. However, 300 μM PFOS was also cytotoxic to the H295R cells (average cell viability 38.2%, Figure 3F). A decrease in viability was also observed in the AR CALUX cells at 100 μΜ (corresponding to the 300 μΜ H295R treatment); however, the induction of steroid hormone biosynthesis at the previous concentration (100 μΜ for H295R cells) is unlikely to be related to cytotoxicity, both because the induction begins at a lower concentration, and because any cytotoxicity would be expected to decrease the signal in the H295R assay. Instead, repeated empirical observations demonstrate an increasing signal. It should also be noted that the viabilities of the CALUX cell lines are unaffected by the 100 μΜ PFOS H295R treatment (30 μΜ final in the CALUX wells), owing to the dilution of the H295R medium into the CALUX cells, suggesting that the hormone quantification is reliable. Altogether these findings indicate that the induction of steroidogenesis by 100 μΜ PFOS can be quantified, but that the magnitude of the induction may vary somewhat between experiments as a borderline effect on cell viability of H295R cells cannot always be excluded. To compensate for this, forskolin was also included as a positive control for induction at a concentration which induces estrogen, but not androgen, biosynthesis, nor provokes nonspecific effects in the CALUX cells.

Next, the performance criteria for this assay were partly revised to reflect the different nature of the detection system used in this version of the H295R steroidogenesis assay, but with reference to the original criteria established during the OECD validation study (OECD, 2010). All quality measures collected, whether during preliminary investigations, as part of dropped experiments or the tests of the present study were pooled together into boxplots and used to establish or re-establish quality control criteria (Supplementary Figs. 2 and 3). Additional criteria specific to the CALUX assays (Besselink, 2015; OECD, 2016, 2020) were also re-assessed using our internal historical dataset and added as well. The final criteria are summarized in Tables 1–3. Generally, tests out of compliance with these requirements were excluded from the dataset and repeated, unless the values of the criteria remained within 10% of the criteria threshold and/or the findings confirmed those observed during additional valid experiments.

**Table 1.** Performance criteria for hormone measurement systems

	Estrogens	Androgens	Anti-Androgens
LOQ (%RI)*^a^*	≤20	≤15	≤15
Hormone production in the solvent control*^b^*	≥2.5* LOQ	≥5* LOQ	≥5* LOQ
Hormone production in the +H295R cells solvent control*^c^*	20–80% of the maximum E2 production in the standard curve	20–80% of the maximum DHT production in the standard curve	40–160% of the 0.3 nM DHT agonist-only wells
Sigmoidal curve fit for reference compound (R^2^)*^d^*	0.95	0.96	0.96
AC_50_ of reference compound (M)*^d^*	6.5E−12 to 3E−11	1.2 E−10 to 4.3E−10	2.4E−7 to 1.4E−6
Induction/inhibition factor (IF) for standard curve*^d^^,^^e^*	8	18	10
Z-factor for standard curve*^d^^,^^f^*	0.5	0.6	0.5

a LOQ=(average % relative induction of vehicle control)+10×SDs.

b Criteria maintained similar to those suggested by the OECD (2011).

c This criterion was relevant for main experiments but was not always considered for the quality control plates, as the E2 producing capacity of the H295R cells was often lower only 3 passages after thawing.

d Criteria relevant for CALUX (Besselink, 2015; OECD, 2016, 2020).

e $\mathrm{IF}\;=\;\frac{\mu_{\mathrm{RLU}\;\mathrm{positive}}}{\mu_{\mathrm{RLU}\;\mathrm{negative}}}$

f Z-factor=$1-\frac{3*(\sigma_{\;\mathrm{RLU}\;\mathrm{positive}}+\sigma_{\mathrm{RLU}\;\mathrm{negative}})}{\left|\mu_{\mathrm{RLU}\;\mathrm{positive}}-\mu_{\mathrm{RLU}\;\mathrm{negative}}\right|}$

**Table 2.** Performance criteria for control substances

	Estrogens	Androgens	Anti-androgens
Induction (Forskolin, 1 μΜ)	≥150% of DMSO control	—	—
Induction (PFOS, 100 μΜ)	—	≥115% of DMSO control*^a^*	≥115% of DMSO control^a^
Inhibition (Prochloraz, 1 μΜ)*^b^*	≤50% of DMSO control	≤50% of DMSO control	≤50% of DMSO control
Cell viability of menadione	<20% in the H295R plate		

a PFOS was used as an alternative control to monitor the performance of the AR CALUX assay, due to nonspecific interference of forskolin with AR CALUX cells. As PFOS was not a strong inducer, a lower threshold was set to distinguish induction.

b Criteria maintained similar to those suggested by the OECD (2011).

**Table 3.** Acceptable ranges and/or variation (%) for H295R assay test plate parameters

	Estrogens	Androgens	Anti-Androgens
Within plate CV of DMSO control*^a^*	≤30%	≤30%	≤30%
Between plate CV of DMSO control for triplicates of the same chemical*^a^*	≤30%	≤30%	≤30%

a Criteria maintained similar to those suggested by the OECD (2011).

To verify the performance of H295R cells after recovery from cryopreservation and their compliance with the above-mentioned quality data, a quality control plate was performed after thawing a new cryovial. An example of the data from one of these control plates is given in Supplementary Figure 4. Both forskolin (Supplementary Figure 4C) and PFOS (Supplementary Figure 4E) increased estrogen biosynthesis, whereas only exposure to PFOS led to an apparent increase in androgens. Prochloraz caused a concentration-dependent decrease in both hormones (Supplementary Figure 4A). The cytotoxicity in the middle concentration of forskolin was not considered relevant due to a lack of concentration-response (Supplementary Figure 4D). The marginal cytotoxicity after exposure to 100 μΜ PFOS (74%, Supplementary Figure 4F) was considered acceptable, as it is both consistently observed and does not correlate with measured androgen activity (Supplementary Figs. 5 and 6). Together these results verify that the H295R cells are responding as expected and can be of use for further experiments.

**Figure 4.**
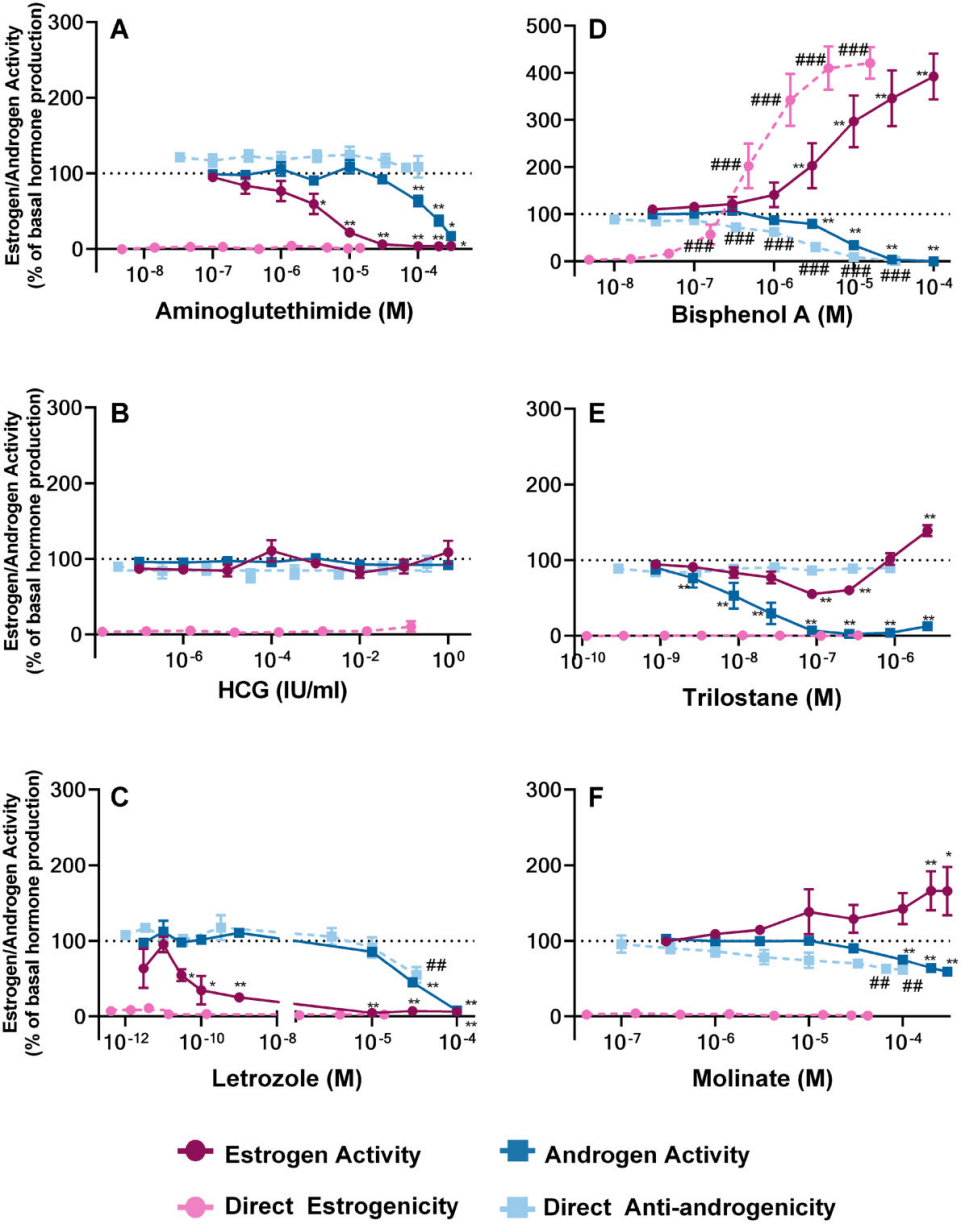
**Apparent induction or inhibition of estrogen and androgen synthesis in the presence (estrogen/androgen activity) and absence (direct estrogenicity/anti-androgenicity) of H295R cells for chemicals of the validation set where cytotoxicity was not evident**. Activities were quantified in response to aminoglutethimide (A), HCG (B), letrozole (C), bisphenol A (D), trilostane (E), and molinate (F) treatment. Concentrations are reported in M for A, C–F and in IU/ml for HCG (B) (1 IU/ml=3.9×10^−6^ M). Direct estrogenicity (ERα agonism, 0%=no activity) and anti-androgenicity (AR antagonism, 100%=no activity) were also measured. Reported concentrations reflect either the final well-concentration in the H295R assay (estrogen activity and androgen activity) or the mean final concentration in the CALUX wells (direct estrogenicity and direct anti-androgenicity). The graphs depict the results of 3 biologically independent experiments made up of 3 technical replicates (*n*=9 when pooled for statistical analysis, with the exception of letrozole, where *n*=6 for the following concentrations: 3×10^−12^, 3×10^−11^, and 10^−10^ M and aminogluthethimide where *n*=6 for 3×10^−4^ M and *n*=3 for 10^−7^ M). Each point represents the mean of means ± SEM. Significance was evaluated by a Kruskal-Wallis test, followed by a Mann-Whitney *U* test for multiple comparisons. The response at each concentration was compared with basal H295R hormone synthesis when treated with 0.1% DMSO vehicle (estrogen or androgen activities; *.01<*p*≤.05, **.001<*p*≤.01, ***.0001<*p*≤.001, ****p≤.0001) or the corresponding sham treatment (direct estrogenicity and (anti-)androgenicity; #.01<*p*≤.05, ##.001<*p*≤.01, ###.0001<*p*≤.001, ####*p*≤.0001).

**Figure 5.**
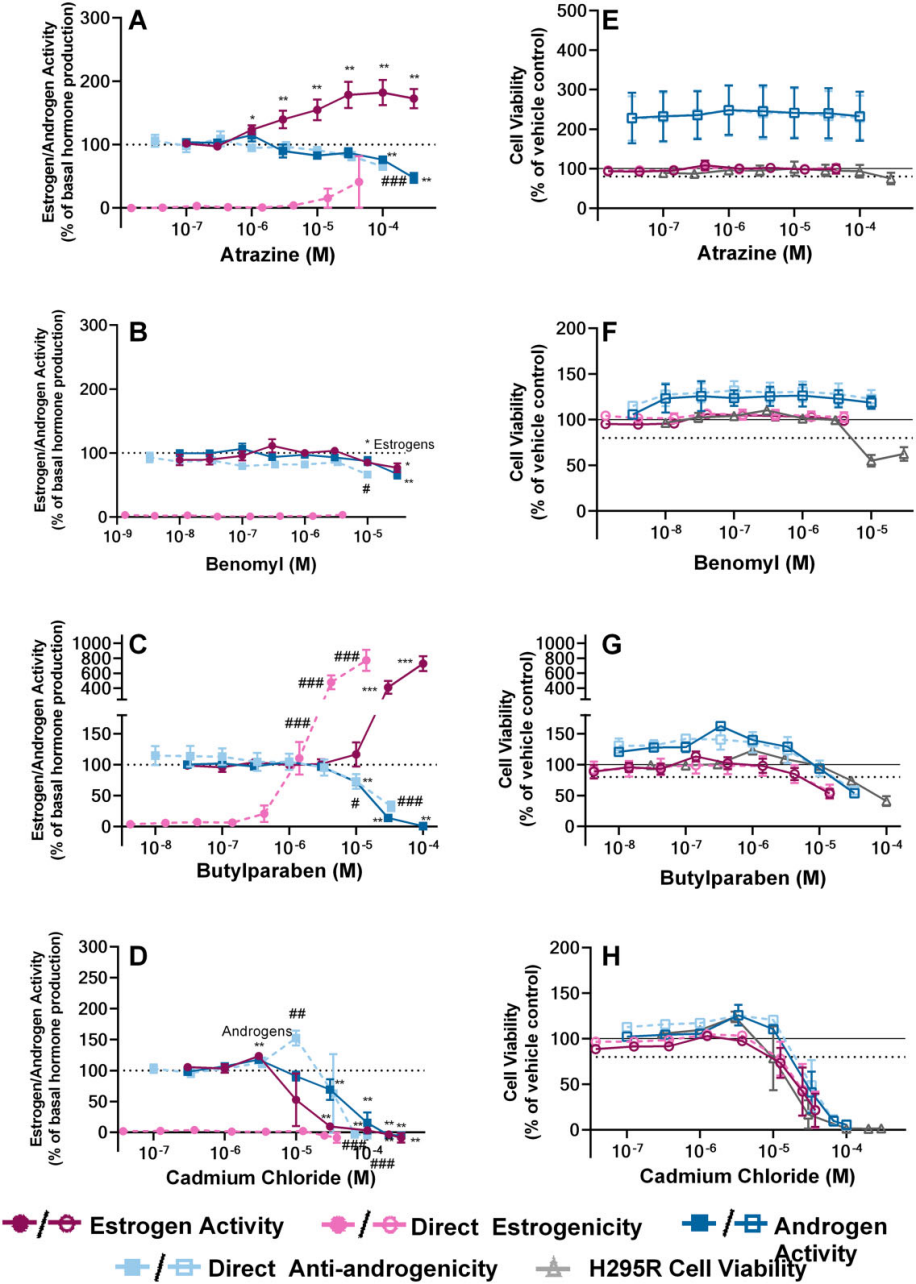
**Results of the H295R steroidogenesis assay for chemicals leading to reduced cell viability**. A–D, Apparent induction or inhibition of estrogen and androgen synthesis in the presence (estrogen/androgen activity) and absence (direct estrogenicity/anti-androgenicity) of H295R cells were quantified in response to treatment with atrazine (A), benomyl (B), butylparaben (C), and cadmium chloride (D). Direct estrogenicity was assessed in an agonistic mode (0%=no activity), whereas direct androgenicity in an antagonistic mode (100%=no activity). Additional same-well cell viability measurements were also made for the H295R cells, as well as the ERα and AR U2OS CALUX cells (Panels E–H). Reported concentrations reflect either the final well-concentration in the H295R assay (estrogen activity, androgen activity and H295R cell viability) or the mean final concentration in the CALUX wells (direct estrogenicity, direct anti-androgenicity and CALUX cell viability). The graphs depict the results of 3 biologically independent experiments made up of 3 technical replicates (*n*=9 when pooled for statistical analysis). Each point represents the mean of means ± SEM. Significance was evaluated by a Kruskal-Wallis test, followed by a Mann-Whitney *U* test for multiple comparisons. The response at each concentration was compared with basal H295R hormone synthesis when treated with 0.1% DMSO vehicle (estrogen or androgen activities; *.01<*p*≤.05, **.001<*p*≤.01, ***.0001<*p*≤.001, ****p≤.0001) or the corresponding sham treatment (direct estrogenicity and anti-androgenicity; #.01<*p*≤.05, ##.001<*p*≤.01, ###.0001<*p*≤.001, ####*p*≤.0001).

Of the 12 chemicals used in this study (Figs. 3–5), no cytotoxicity was observed in the tested range after treatment with 8: forskolin, prochloraz (Figure 3), aminoglutethimide, trilostane, human chorionic gonadotropin (HCG), letrozole, molinate, and bisphenol A (BPA) (Supplementary Figure 7). As a result, any observed activities of these chemicals can be attributed either to steroidogenesis or direct effects on the receptor. Of these noncytotoxic substances, only HCG (Figure 4B) had no effect on H295R androgen and estrogen steroidogenesis, nor any direct effect on the androgen and estrogen receptor. Note that the concentration range used for this study was lower than the one of the OECD (2010). Nevertheless, these results are not expected to change, as the H295R cell line was validated for the detection of effects that alter steroidogenesis downstream of the gonadotropin hormone receptors (OECD, 2010), where HCG exerts no effect (Hecker *et al.*, 2011).

Steroidogenic effects were correctly identified for aminoglutethimide (Figure 4A), prochloraz (Figure 3A), and trilostane (Figure 4E). In the case of aminoglutethimide and prochloraz a clear concentration-dependent reduction in the levels of both assessed hormone-families was observed. The lowest effect concentration (LOEC) for aminoglutethimide was equal to 3 μΜ for estrogens and 100 μΜ for androgens; for prochloraz LOECs equal to 0.03 μΜ were observed for both estrogens and androgens (the lowest concentration tested). Trilostane exposure led first to a concentration dependent decrease (LOEC = 0.1 μΜ) in H295R estrogen production, followed by a concentration dependent increase in estrogens. By contrast, only a decrease in androgen biosynthesis was evident (LOEC = 0.003 μΜ).

Partial detection of steroidogenic effects was seen for forskolin (Figure 3B), molinate (Figure 4F), and letrozole (Figure 4C). In the case of forskolin, effects on androgen synthesis could not be confirmed using the AR CALUX, whereas the expected tendency was observed for estrogens (LOEC = 0.3 μΜ), as previously mentioned. Molinate statistically significantly increased estrogen synthesis at and above the 200 μΜ LOEC. Apparent androgen synthesis was statistically significantly decreased at the 3 maximal concentrations of molinate treatment (LOEC = 100 μΜ H295R treatment). Direct receptor antagonism was also observed, though it was only statistically significant at the 2 maximal concentrations (LOEC = 67 μΜ in the AR CALUX cell medium, equivalent to 200 μΜ in the H295R cells). To verify that the apparent decrease in androgen activity is in fact due to direct anti-androgenicity, we plotted the means for each biological replicate of each condition separately, without taking dilution into account (Supplementary Figure 8A). As the replicates of androgen activity and direct anti-androgenicity were indiscernible, the apparent decrease in androgen synthesis was judged to be the result of anti-androgenicity; no effect on androgen synthesis could be determined at those molinate concentrations.

A similar effect of letrozole on apparent androgen synthesis was also observed. Both decreasing apparent androgen synthesis and direct receptor antagonism was observed at the 2 maximal treatment concentrations; however, only the androgen levels at the maximum 2 concentrations (LOEC = 30 μΜ H295R treatment) and the direct receptor antagonism at the top concentration (LOEC = 33 μΜ in the AR CALUX, equivalent to 100 μΜ H295R treatment) were statistically significant. Plots of the individual biological replicates (Supplementary Figure 8B) indicated that in this case both activities might be present at the same concentrations. This is somewhat supported by a lack of overlap between the 95% confidence intervals of these activity measurements (35–56% for androgenic activity and 64–118% relative induction for direct anti-androgenicity at 30 μΜ H295R treatment). However, this is smaller than 10%, well within normal variation for this assay, so the biological relevance of this difference is unclear. As a result, we decided to follow a conservative approach and characterize letrozole as negative for effects on androgen synthesis. However, letrozole clearly caused an anticipated decrease in estrogen levels at 30 pM, making it endocrine active at doses well below those which might affect androgen synthesis.

Lastly, no apparent steroidogenic effects could be identified for BPA (Figure 4D). Direct agonism of the estrogen receptor and direct antagonism on the androgen receptor were observed with LOECs of 0.16 and 0.3 μΜ, respectively. Consequently, the chemical was characterized as negative for effects on steroidogenesis, although the possibility of a steroidogenic effect of BPA at concentrations above these receptor-LOECs cannot be definitively excluded.

By contrast, cytotoxicity was observed in at least one concentration of the remaining 4 substances: atrazine, benomyl, butylparaben, and cadmium chloride; this also must be accounted for in test result interpretation. For atrazine, the marginal cytotoxicity at the highest concentration of H295R cells (300 μΜ, Average viability = 75%, Figure 5E) did not interfere with the detection of effects on steroidogenesis. In detail, a concentration-dependent increase was observed at 1 μΜ for estrogens, as expected. A concentration dependent decrease in apparent androgen biosynthesis was also observed and statistically significant at the maximum 2 concentrations (LOEC = 100 μΜ); however, direct receptor antagonism was also observed at the same nominal concentration on CALUX cells (Figure 5A) and plotting the individual replicates revealed that the 2 effects are indiscernible (Supplementary Figure 8C). This co-occurrence prevents the conclusive determination of whether there is a steroidogenic effect at these concentrations and therefore has resulted in the characterization of this chemical as negative for effects on androgen biosynthesis.

Exposure of H295R cells to benomyl led to a concentration-dependent decrease in the levels of estrogens and a decrease at the highest concentration for androgens (Figure 5B). However, cytotoxicity was also observed (Figure 5F), but only in H295R cells; U2OS CALUX cell viability was within normal levels. Thus, this reduced estrogen and androgen production was judged to be linked to the reduced H295R cell viability; therefore, benomyl was characterized as negative for effects on steroidogenesis in the present assay. Nevertheless, as the reduction in androgen activity of the sham wells was accompanied by absence of cytotoxicity, benomyl was evaluated as positive for anti-androgenic effects. Cytotoxicity was also observed in all cell lines at the maximum concentrations of butylparaben (Figure 5G), and in multiple concentrations of cadmium chloride (Figure 5H). The presence of this cytotoxicity, together with the strong direct estrogenic and anti-androgenic effects of butylparaben (Figure 5C), also led to the characterization of this chemical as negative for perturbations in steroidogenesis in this test, although a secondary steroidogenic effect with a higher LOEC than the one observed for direct receptor activities cannot be excluded. Similarly, cadmium chloride, a known cytotoxic agent in various cell lines (Fotakis and Timbrell, 2006; Marin-Kuan *et al.*, 2017; Romero *et al.*, 2003) showed a reduction in steroid hormone production which can be attributed to reduced cell viability (Figure 5D and H).

To summarize assay performance, several comparisons were performed between the data of the current study and existing literature, starting with a comparison with the findings of the OECD validation of the H295R assay (Hecker *et al.*, 2011). For the comparison with *in vitro* data the following chemicals were used: aminoglutethimide, atrazine, benomyl, BPA, butylparaben, forskolin, HCG, letrozole, molinate, prochloraz, trilostane. For the comparison with *in vivo* data the following chemicals were used: aminoglutethimide, atrazine, benomyl, BPA, butylparaben, HCG, letrozole, molinate, prochloraz, trilostane. Forskolin was omitted due to lack of *in vivo* data, whereas PFOS and cadmium chloride were omitted from all the comparisons as they were not part of the original OECD validation, as described under Materials and methods section. For detailed classification of chemicals please consult Supplementary Table 2.

**Figure 6.**
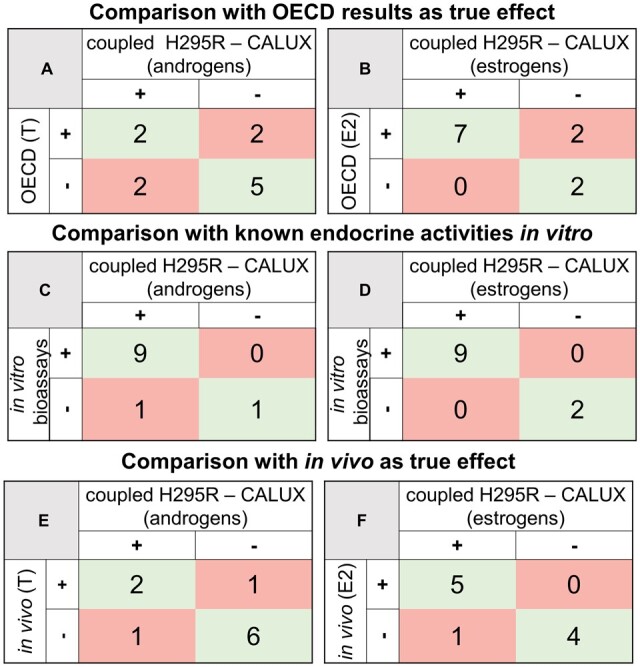
**Confusion matrices for the comparison of the results obtained in the H295R-CALUX assay.** Comparison to the results of the OECD validation of the H295R assay for steroidogenesis of T (A) and E2 (B). Comparison to the OECD results plus previously reported *in vitro* receptor-mediated endocrine activities relating to androgens (C) and estrogens (D). Comparison to *in vivo* data for testosterone (E) and estradiol (F) biosynthesis.

The combined H295R-CALUX assay correctly classified all 5 negative substances for alterations to androgen production: atrazine, benomyl, butylparaben, HCG, and molinate. Moreover, 2 out of the 4 positive chemicals, prochloraz and aminoglutethimide were correctly identified. The remaining 2 positive chemicals in the OECD study, namely BPA and letrozole, were characterized as false negatives. The effects of forskolin and trilostane were in the opposite direction and these substances were characterized as false positives, resulting in moderate values for sensitivity, specificity, and accuracy, equal to 0.50, 0.71, and 0.64, respectively (Figure 6A and Table 4).

In the case of estrogens, 7 chemicals were correctly classified as positive (prochloraz, forskolin, aminoglutethimide, trilostane, atrazine, letrozole, and molinate) or negative (HCG and benomyl) for effects on steroidogenesis. BPA and butylparaben were misclassified as negatives, leading to values equal to 0.78 and 0.82 for sensitivity and accuracy, respectively (Figure 6B and Table 4). Specificity was not calculated for the estrogens due to the low number of true negatives.

While these sensitivities of 0.50 and 0.78 are only moderately good on their own, notably, the reduction in these values is mainly linked to relatively high false-negative rates due to the presence of direct receptor (ant-)agonism, which are equally important endocrine activity modalities. To take this into account and better characterize the performance of the coupled assay to predict leading endocrine effects, a second set of confusion matrices was constructed in which the results of the combined H295R-CALUX were compared with existing *in vitro* measurements of steroidogenic and receptor-mediated endocrine activities (Figs. 6C and 6D). Consequently, the specificity and accuracy of the assay for androgens rose to 1.00 and 0.91, respectively, as the AR antagonist BPA previously identified as a false-negative, and forskolin and trilostane as contra-directional modifiers of steroidogenesis, have all now been re-categorized correctly as endocrine active substances (Table 4 and Figure 6C) (Hecker *et al.*, 2011; Wang *et al.*, 2014b). The remaining AR antagonist, letrozole, is now characterized as a false positive due to previously unrecorded antagonism of the androgen receptor. The improvement in the specificity and accuracy of the coupled H295R-CALUX assay for estrogens was even more marked than for androgens; both values are now estimated to be 1.00, as BPA and butylparaben, the 2 ERα agonists previously identified as false-negatives due to their receptor-mediated leading activity, were re-categorized as true positives in the new confusion matrix (Table 4 and Figure 6D) (van der Burg *et al.*, 2015; Wang *et al.*, 2014b). Note that neither specificity could be estimated due to the small number of truly negative substances tested.

However promising, these comparisons do not provide any insight into the ability of the coupled H295R-CALUX assay to predict relevant effects *in vivo*. In order to determine the predictive value of the linked assay, a third pair of confusion matrices (Figs. 6E and 6F) compared the data from the current study with the *in vivo* results summarized by Hecker *et al.* (2011).

The coupled H295R-CALUX steroidogenesis assay correctly classified 2 steroidogenic (prochloraz and trilostane) and 6 negative substances (atrazine, benomyl, BPA, butylparaben, HCG, and molinate) for alterations to T biosynthesis. A further 2 substances were falsely identified as false-negative and false-positive results: letrozole and aminoglutethimide, respectively (Figure 6E). This performance was slightly better than that of the original version of the H295R assay in the validation study, where only 1 positive (prochloraz) and 5 negative substances (atrazine, benomyl, butylparaben, HCG, and molinate) were predicted correctly; a further 4 negative substances (aminoglutethimide, BPA, letrozole, trilostane) were misjudged positive in this version of the assay. Consequently, the calculated specificities and accuracies were 0.86 and 0.80 for the coupled H295-CALUX assay to 0.55 and 0.60 for the Hecker *et al.* H295R protocol (2011). Sensitivity was not calculated due to the low number of true positive samples in this test (Table 5).

Similarly, the combined assays correctly classified 5 steroidogenic (aminoglutethimide, atrazine, letrozole, prochloraz, trilostane) and 4 negative substances (benomyl, BPA, butylparaben, and HCG) for alterations to E2 biosynthesis (Figure 6F). A further substance, molinate, was misidentified as inducer of estrogen synthesis. Again, this performance was slightly better than that of the original H295R protocol where only 4 positive and 2 negative substances were predicted correctly (aminoglutethimide, atrazine, letrozole, prochloraz, benomyl, and HCG); a further 4 negative substances (BPA, butylparaben, molinate, trilostane) were misjudged positive. Consequently, the calculated sensitivities, specificities, and accuracies for estrogen synthesis were 1.00, 0.80, and 0.90 for the coupled H295-CALUX assay to 1.00, 0.33 to 0.60 for the Hecker *et al.* H295R protocol (2011) (Table 5).

Altogether, the findings from the 3 pairs of confusion matrices suggest a relevance of the coupled assay for use as a single test for substances with anti-androgenic, estrogenic and/or steroidogenic endocrine activities. Importantly, despite the improved metrics of the H295R-CALUX versus those of the OECD, it has to be acknowledged that both studies have limitations and strengths when it comes to predicting true *in vivo* effects, with some of them being common in both assays due to the use of H295R cells as a model for steroidogenesis.

## Discussion

Endocrine active substances may interact with the synthesis and action of hormones, which are key events of toxicological interest requiring the development of robust *in vitro* screening methods. The present study explored ERα and AR CALUX bioassays as an alternative detection system for the original H295R steroidogenesis assay, that can simultaneously provide information on direct receptor effects.

Coupling the H295R assay to the CALUX bioassays was not without difficulties and drawbacks. The developed assay did not detect the expected increase in androgen levels after exposure to forskolin, a steroidogenesis inducer and established positive control. Steroidogenesis induction usually takes place through various signaling pathways (OECD, 2011) that can also be present in other cell systems. Forskolin activates the cAMP pathway (Hilscherova *et al.*, 2004) and mediates the rate-limiting mitochondrial cholesterol transport step in steroidogenesis (Liu *et al.*, 2006). The cAMP second messenger system is also relevant to many biological pathways and forskolin may have interfered via other mechanisms, potentially including but not restricted to dephosphorylation of the AR receptor (Blok *et al.*, 1998). This may have interfered with the detection of changes in androgen levels by the AR CALUX cells. Given that androgen increases by AR CALUX cells are reportedly well-detected (Sonneveld *et al.*, 2006), several additional chemicals were tested for their potential to increase both estrogen and androgen production: tricresyl phosphate (Hecker *et al.*, 2011), sildenafil (Kang *et al.*, 2013), perfluorooctanoic acid, PFOS (Kraugerud *et al.*, 2011; Pinto *et al.*, 2018; van den Dungen *et al.*, 2015), clotrimazole (Hinfray *et al.*, 2011), malathion (Taxvig *et al.*, 2013), and microcystin-LR (Hou *et al.*, 2018) (data not shown). However, the experiments with forskolin revealed that the mechanism of induction needs to be more specific to steroidogenesis than just general interference with second messenger pathways. As this kind of specificity seems to be rare, it is not surprising that only PFOS apparently induced androgen production. Cytotoxicity was often observed in H295R cells at PFOS concentrations equal to or greater than 100 μΜ (Supplementary Figure 5). This was lower than the ≥300 μΜ previously reported (Kraugerud *et al.*, 2011; van den Dungen *et al.*, 2015), a difference explained by the more sensitive, luminescence-based assay for cell viability (Riss *et al.*, 2013, 2016). Lacking a better alternative, 2 positive controls for hormone induction were included in each experiment: the weak inducer PFOS (100 μΜ) for androgen production, with lower acceptance thresholds, and forskolin (1 μΜ) for estrogen synthesis.

Another challenge in the H295R-CALUX assay was the variation in the basal estrogen levels produced by the H295R cells. This issue has been previously reported (Hecker *et al.*, 2006); the OECD guidance suggested using H295R cells between passages 4 and 10 after a single freeze-thaw cycle. In the H295R-CALUX, however, we continued to observe this variation even in cells cultured according to the guidelines, which invalidated some test runs due to estrogen levels outside of the quantifiable range. This augmented variability may be attributed to the increased responsiveness of the CALUX-detection system to small changes in hormone levels. To reduce the risk of invalidation, multiple dilutions into the ERα CALUX were included. On a longer-term basis, this issue could be minimized by reconstructing the ERα CALUX cell line to select a larger dynamic range of response from the reporter gene.

Lastly, for chemicals with both reported steroidogenic and direct receptor activities, the coupled H295R-CALUX assay identified only the leading modality, thereby preventing the detection of steroidogenic effects in cases where receptor activity occurs at the same or lower concentration than the expected steroidogenic activity. Consequently, BPA and letrozole were misclassified as negative for effects on androgen biosynthesis and BPA and butylparaben for effects on estrogen biosynthesis. Nevertheless, when these data are re-classified based on the presence or absence of any endocrine modality, concordant results were observed for the leading activity of almost all substances: prochloraz, forskolin, aminoglutethimide, trilostane, atrazine, molinate, bisphenol A, butylparaben, benomyl (all endocrine active), and HCG (for which H295R cells are known to be unresponsive). The only exception was letrozole for which a previously unreported antagonism of the androgen receptor was observed. Letrozole is a member of the azole family of aromatase inhibitors, many members of which are known androgen receptor antagonists (Roelofs *et al.*, 2014). Therefore, the measured direct activity is likely indicative of a genuine *in vitro* activity in addition to letrozole’s steroidogenic effects. Nonetheless, the ability of letrozole to perturb estrogen biosynthesis was correctly identified.

Altogether, the moderate sensitivities, specificities, and accuracies reported for the coupled H295R-CALUX assays, based on comparisons with the OECD validation dataset, substantially undervalue the assay’s overall utility for the identification of samples with endocrine activity. Depending on the testing context, the H295R-CALUX could be used to achieve different outcomes. As part of a 2-phase testing strategy to increase throughput, it could be used as a first screen for estrogenic, anti-androgenic, and steroidogenic activities with secondary individual-modality testing only performed for positive substances. In certain regulatory contexts, the presence of endocrine activities may need to be excluded (eg, in the European Union for many uses). There, the coupled H295R-CALUX could be used as part of a testing strategy enhance decision making (eg, between candidate molecules early in development) or even for regulatory decision making in contexts where *in vivo* testing is either not legal (eg, cosmetics in Europe) or not practical (eg, packaging migrates or food extracts).

Despite the challenges associated with coupling 2 bioassays, the H295R-CALUX had several benefits. Importantly, it was able to overcome the existing limitations in the quantitation of estrogen hormone levels. Consequently, lower LOEC values were observed for 3 aromatase inhibitors known to reduce estrogen biosynthesis: prochloraz, aminoglutethimide, and letrozole. Notably, the latter LOEC was dramatically reduced; at the 100 pM LOEC reported by Hecker *et al.* (2011), H295R letrozole estrogen production was still <50% of basal synthesis using the CALUX detection system. By contrast, the LOECs of 2 of 4 inducers of estrogen biosynthesis (atrazine and trilostane) were identical to those previously described; the exceptions were forskolin and molinate which failed to attain statistical significance at earlier concentrations, possibly due to the increased variability among the 3 biological experiments. These results suggest that the previous LOECs reported for the 3 aromatase inhibitors may be at least partly attributed to the proximity of H295R basal E2 production to the limits of immunoassay and LC-MS/MS E2 quantitation and highlight the need for more sensitive detection methods, such as the CALUX.

Moreover, the H295R-CALUX managed to detect both inhibition of estrogen biosynthesis (also observed *in vivo*), and a subsequent induction with increasing trilostane concentration (Table 7). This dual behavior has also been reported by Jumhawan *et al.* (2017), who preconcentrated the cell culture supernatant using solid phase extraction and analyzed it by GC-MS. And because the coupled H295R-CALUX assay did not use antibodies, the well-described cross-reactivity between trilostane and testosterone-antibodies (Hecker *et al.*, 2011) was avoided, thereby improving its androgen-biosynthesis LOEC and reversing the direction of effects to agree with those observed *in vivo*. Taken together with the above-mentioned reduction in some LOECs, the ability of the coupled H295R-CALUX to more closely model the *in vivo* pharmacology of trilostane supports the use of the proposed detection system in some contexts.

The present study also utilized a sensitive assay for the determination of cell viability. Because the luminescence-based measure of reducing potential used is as sensitive to cell viability alterations as the luciferase reporter gene of the CALUX assay (Marin-Kuan *et al.*, 2017), deconvoluting whether reduced CALUX activity is related to viability or receptor-mediated changes is simple. This contrasts with our laboratory’s previous experience with formazan-based assays (like the MTT) which lag behind the CALUX luciferase activity in detecting cytotoxicity. Additionally, since the RT-Glo assay is nonlytic, both viability and reporter-gene activity can be determined in the same wells, removing the need for parallel test plates. Tests of cadmium chloride illustrated the utility of assessing cytotoxicity alongside the effect of interest. As expected, the chemical’s leading activity was cytotoxicity, in line with previous results (Fotakis and Timbrell, 2006; Marin-Kuan *et al.*, 2017; Romero *et al.*, 2003), which prevented the detection of its previously reported steroidogenic effects (Das and Mukherjee, 2013; Lau *et al.*, 1978).

The H295R-CALUX assay offers other additional benefits. A denser 96-well format enables testing of more concentrations of a single chemical, consumes less sample, and allows inclusion of control wells in every test substance plate, for a more robust and higher-quality assay design. Alterations to hormone levels are also easily quantified without prior extraction or concentration steps commonly used (Haggard *et al.*, 2018; Hecker *et al.*, 2011; Jumhawan *et al.*, 2017; Karmaus *et al.*, 2016; Nakano *et al.*, 2016; Nielsen *et al.*, 2012; Rijk *et al.*, 2012; van den Dungen *et al.*, 2015). Automation of the coupled H295R-CALUX is possible in a manner similar to other cell-based assays. And finally, the addition of the direct receptor activities means that one test protocol can be used for multiple endocrine modalities. With progesterone (PR) and glucocorticoid (GR) CALUX bioassays already commercially available (Sonneveld *et al.*, 2006, 2011; van der Linden *et al.*, 2008), the present assay could be expanded to include both direct receptor activities and steroidogenic effects for glucocorticoids and prostagens.

Taken together, the results from this proof-of-concept study indicate that the coupled H295R-CALUX assay has promise for the combined testing for effects on steroidogenesis and direct AR- and ERα-mediated activity. Depending on the testing context, once fitness-for-purpose is established, it could potentially be used as part of a multi-phase *in vitro* testing strategy, serve as a prioritization tool to enhance decision making or be applied to regulatory contexts where endocrine activities are unwelcome and higher tier testing is impractical.

## Supplementary Material

kfad052_Supplementary_Data

## Data Availability

Nestlé Research holds a license from BioDetection Systems (Amsterdam, The Netherlands) for the use of the CALUX assay. All other materials used are widely available. Raw data and analysis files supporting the findings of this study are available from the corresponding author upon reasonable request.
