# Supplementary material for: Coupling the H295R with ERα and AR U2OS CALUX assays enables simultaneous testing for estrogenic, anti-androgenic and steroidogenic modalities
Source: Toxicol Sci. 2023 Jun 1;194(2):191–208. doi: 10.1093/toxsci/kfad052 (PMC10375316; doi:10.1093/toxsci/kfad052)
Supplement: kfad052_Supplementary_Data [file kfad052_supplementary_data.docx]

**SUPPLEMENT**

**Coupling the H295R and ERα and AR U2OS CALUX assays enables simultaneous testing for estrogenic, anti-androgenic and steroidogenic modalities**

Martha S. Nikopaschou^a,b^, Alexandre Félix^a^, Julie Mollergues^a^, Gabriele Scholz^a^, Benoit Schilter^a^, Maricel Marin-Kuan^a^ & Karma C. Fussell^a^

**Table of contents**

| Table S1 Sourcing of test chemicals and standards and final treatment concentrations of test chemicals on H295R cells |  | Page 2 |
| --- | --- | --- |
| Figure S1 Q-Q plots and frequency distributions for chemicals analyzed in the present study |  | Pages 3-4 |
| Figure S2 Boxplots depicting the range of the obtained values for the limit of quantification (LOQ), the half maximal effective/ inhibitory concentration (AC50), the induction factor (IF), the Z-factor and the coefficient of determination (R2) for ER CALUX, AR CALUX and anti-AR CALUX |  | Page 5 |
| Figure S3 Boxplots summarizing the estrogen and androgen activity obtained for the positive control chemicals |  | Page 6 |
| Figure S4 Effect of the positive control chemicals used for quality control on H295R-mediated steroidogenesis and cell viability. |  | Page 7 |
| Figure S5 Cell viability data for the positive control chemicals |  | Page 8 |
| Figure S6 Correlation analysis between H295R cell viability & observed Androgen activity (validation experiments only) |  |  |
| Figure S7 Additional same-well cell viability measurements for the H295R cells, as well as the ER and AR U2OS CALUX cells for the chemicals shown in Figure 5 |  | Page 9 |
| Figure S8 Androgen activity and direct anti-androgenicity for the maximal treatment concentrations of molinate, letrozole and atrazine. |  | Page 10 |
| Table S2 Classification of chemicals in confusion matrices |  | Pages 11-12 |
| R -Script for the performance of the Kruskal Wallis analysis, followed by a series of Mann-Whitney U pairwise comparisons |  | Pages 13-17 |

**Table S1 Sourcing of test chemicals and standards and final treatment concentrations of test chemicals on H295R cells**

| Name | CAS number | Supplier Reference | Lot number | Final treatment concentrations (H295R cells, μM) |
| --- | --- | --- | --- | --- |
| 17β- estradiol (E2) | 50-28-2 | Sigma- Aldrich E8875 | SLBP6339V &  SLBT2822 | - |
| Aminoglutethimide | 125-84-8 | Sigma- Aldrich A9657 | BCBN4336V | 0.1-300 |
| Atrazine | 1912-24-9 | Sigma- Aldrich 90935 | BCBS4552V | 0.1 - 300 |
| Bisphenol A | 80-05-7 | Sigma- Aldrich 239658 | MKBS0991V | 0.03 - 100 |
| Benomyl | 17804-35-2 | Sigma- Aldrich 381586 | MKCG8313 | 0.01 - 30 |
| Butylparaben | 94-26-8 | Sigma- Aldrich 54680 | BBCB7392 | 0.03 - 100 |
| Cadmium Chloride | 10108-64-2 | Sigma- Aldrich 202908 | MKBM1769V | 0.3 - 300 |
| Dihydrotestosterone | 521-18-6 | Sigma- Aldrich 10300 | BCB99347V | - |
| Dimethylsulfoxide (DMSO) | 67-68-5 | Sigma- Aldrich D2650 | RNBJ2585,  RNBG3189,  RNBJ0055,  RNBJ6295  RNBJ9739 | - |
| Flutamide | 13311-84-7 | Sigma- Aldrich F9397 | MKBV6955V |  |
| Forskolin | 13311-84-7 | Sigma- Aldrich F6886 | SLBP3308V | 0.01 - 100 |
| HCG | 9002-61-3 | Sigma- Aldrich C1063 | SLBT6639 |  |
| Letrozole | 112809-51-5 | [Sigma](https://nestle.sharepoint.com/teams/NRCChemicalInventory/_layouts/15/listform.aspx?PageType=4&ListId=%7b3dfb33f1-5852-4696-8094-0e7be9b7ed73%7d&ID=120&RootFolder=*)- Aldrich L6545 | 0000122828 | 3*10^-6^- 100 |
| Menadione | 58-27-5 | Sigma- Aldrich M5625 | STBC4225V | - |
| Molinate | 2212-67-1 | Sigma- Aldrich 36171 | BCBW2692 | 0.3-300 |
| Perfluoorooctanesulfonic  acid | 2795-39-3 | Sigma- Aldrich 77282 | BCCC7858 | 0.1 - 300 |
| Prochloraz | 67747-09-5 | Sigma- Aldrich 45631 | SZBD077XV | 0.03 - 4 |
| Trilostane | 13647-35-3 | Sigma-Aldrich SML0141 | 82991 | 8.7*10^-4^ – 2.6 |

**Figure S1 Q-Q plots and frequency distributions for chemicals analyzed in the present study.**
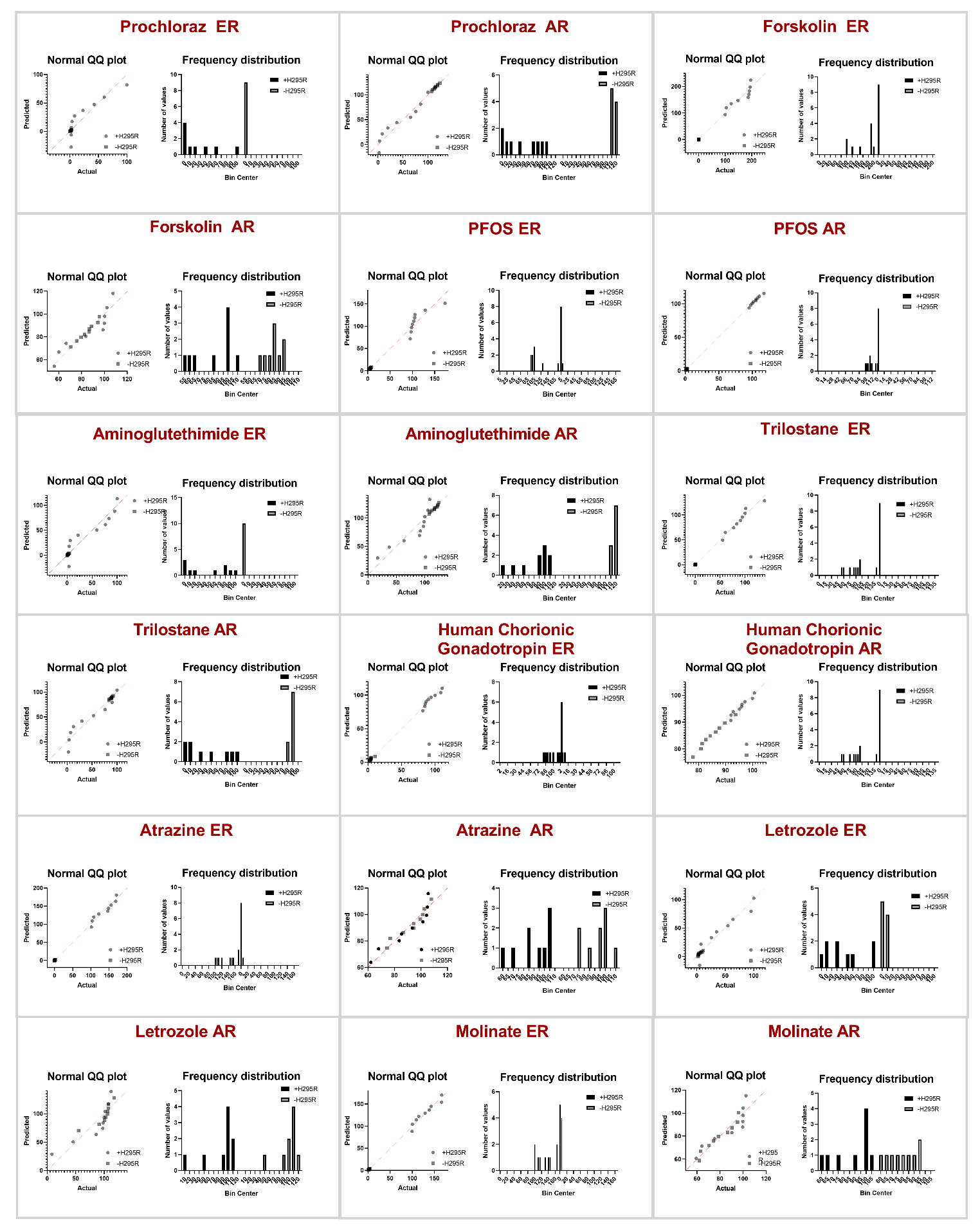


**Figure S1 (continued) Q-Q plots and frequency distributions for chemicals analyzed in the present study.**


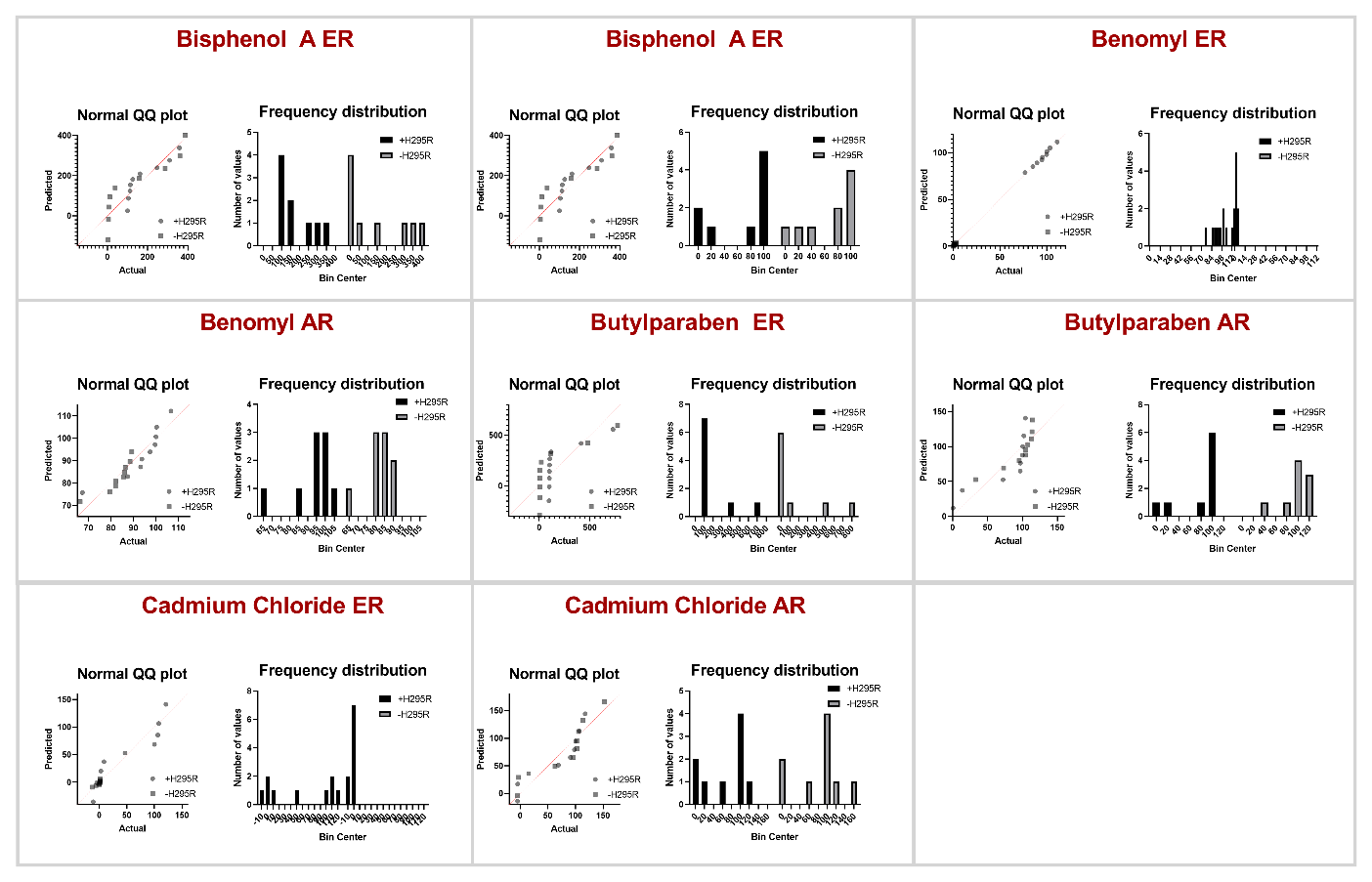


**Figure S2 Boxplots depicting the range of the obtained values for the limit of quantification (LOQ), the half maximal effective/ inhibitory concentration (EC50/IC50), the induction factor (IF), the Z-factor and the coefficient of determination (R2) for ER CALUX, AR CALUX and anti-AR CALUX (preliminary & validation experiments).** The dotted lines represent the accepted performance ranges, also mentioned in Table 1.
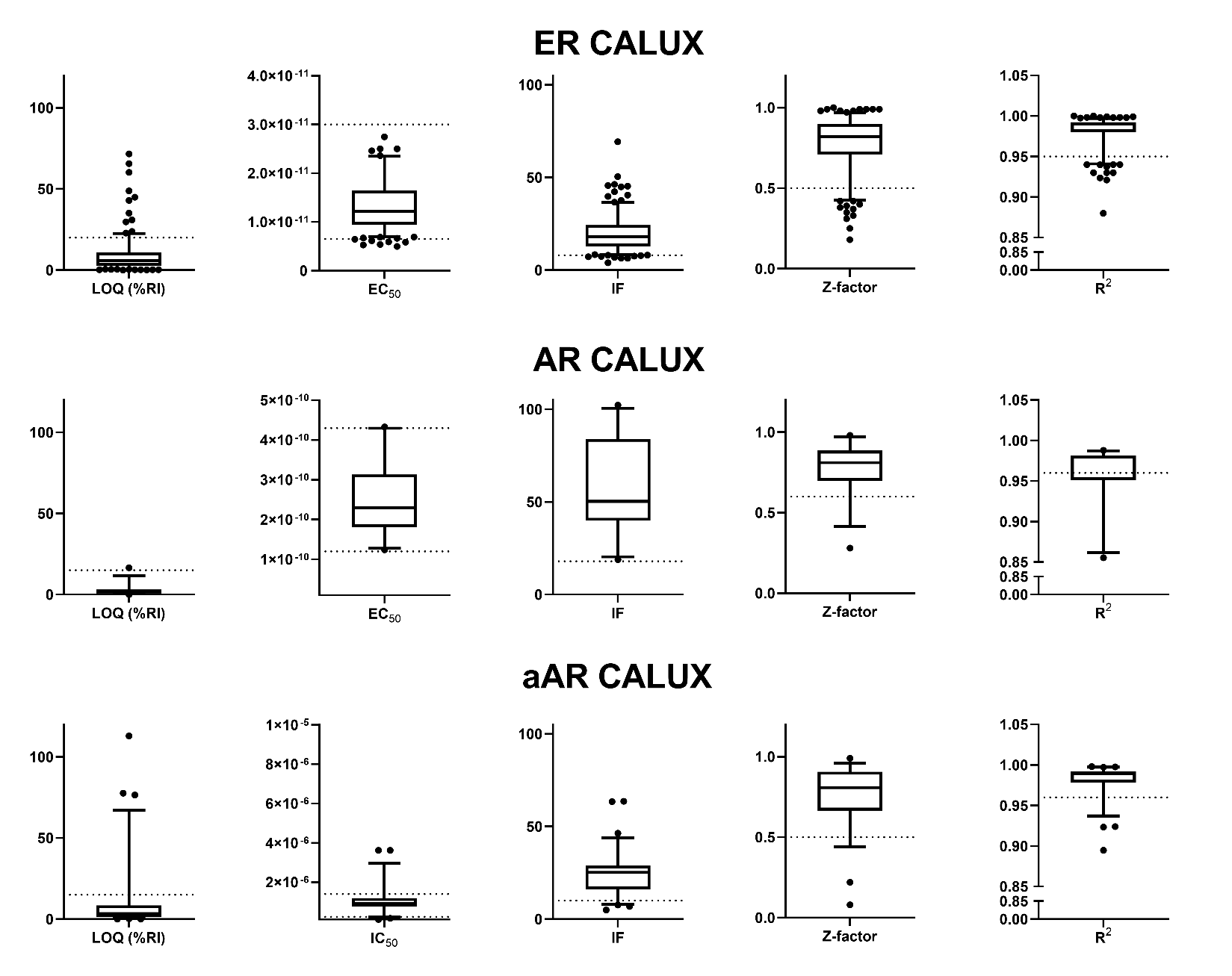


**Figure S3 Boxplots summarizing the estrogen and androgen activity obtained for the positive control chemicals (prochloraz, forskolin & PFOS) throughout this study (preliminary & validation experiments).** Dashed lines are used to mark the 100% activity (activity of the H295R containing wells treated with DMSO only) and dotted lines show the threshold for the performance criteria, summarized in Table 1.*
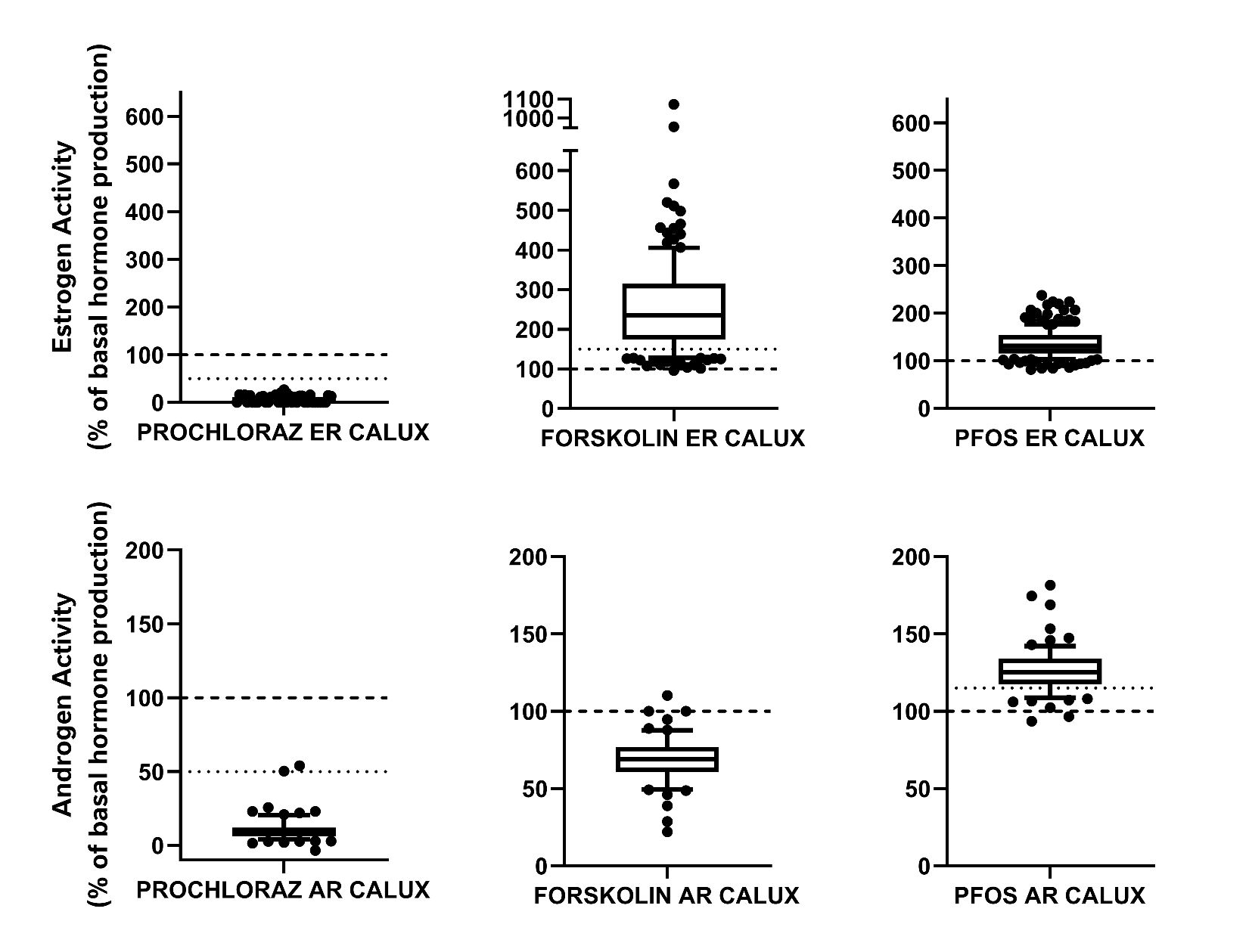
*

**Figure S4 Effect of the positive control chemicals used for quality control on H295R-mediated steroidogenesis and cell viability.**

A-C: Apparent induction or inhibition of estrogen and androgen synthesis in the presence (Estrogen/Androgen Activity) and absence (Direct Estrogenicity/Androgenicity) of H295R cells. Additional same-well cell viability measurements were also made for the H295R cells, as well as the ER and AR U2OS CALUX cells (Panels D-F). Reported concentrations reflect either the final well-concentration in the H295R assay (estrogen activity, androgen activity and H295R cell viability) or the mean final concentration in the CALUX wells (direct estrogenicity and direct androgenicity and CALUX cell viability). The figure shows an example of a single quality control experiment for the evaluation of cell-performance; each data point represents the mean ± the standard deviation of n=3 technical replicates.


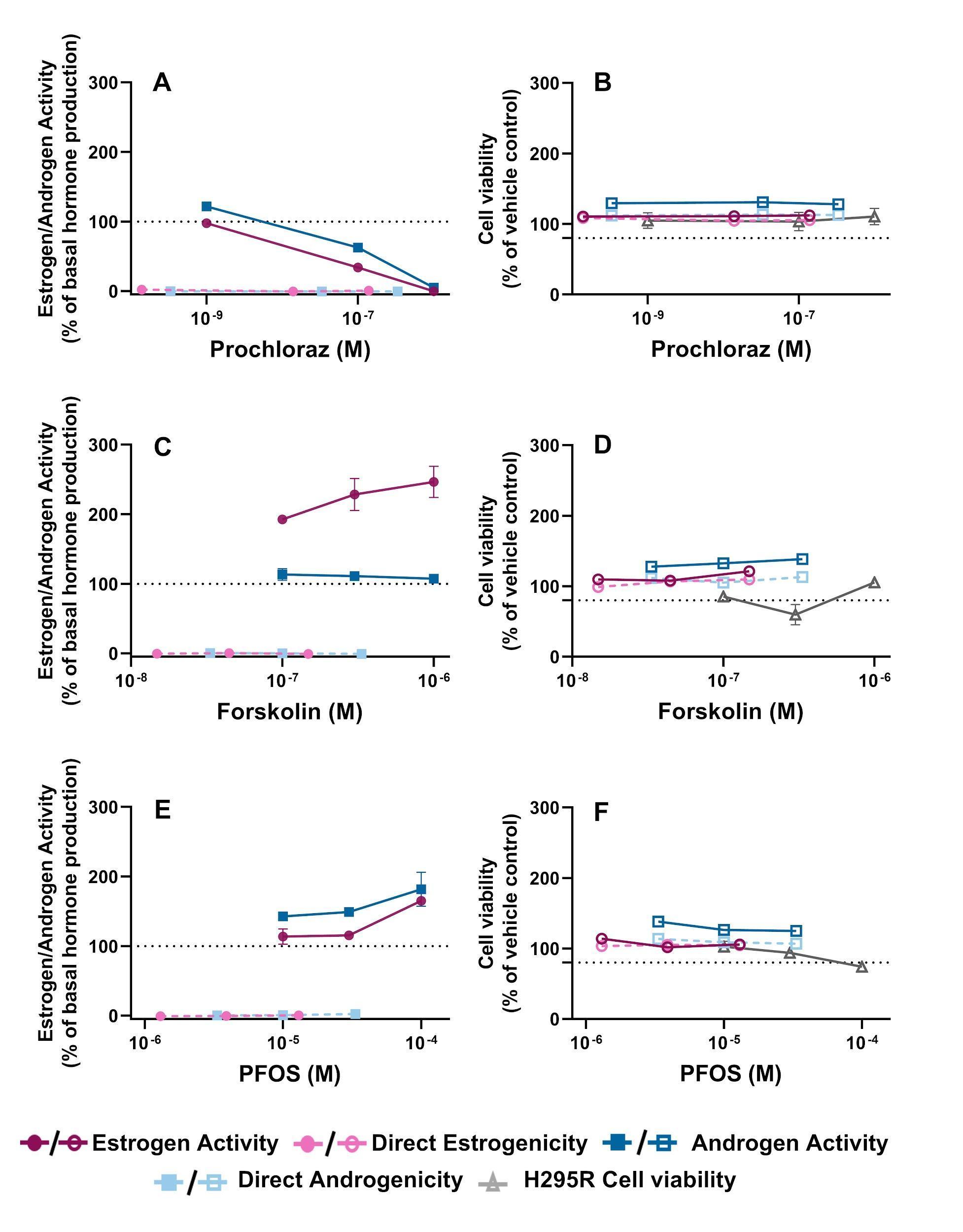


**Figure S5 Cell viability data for the control chemicals (Prochloraz, Forskolin, PFOS & menadione) (validation experiments only).**


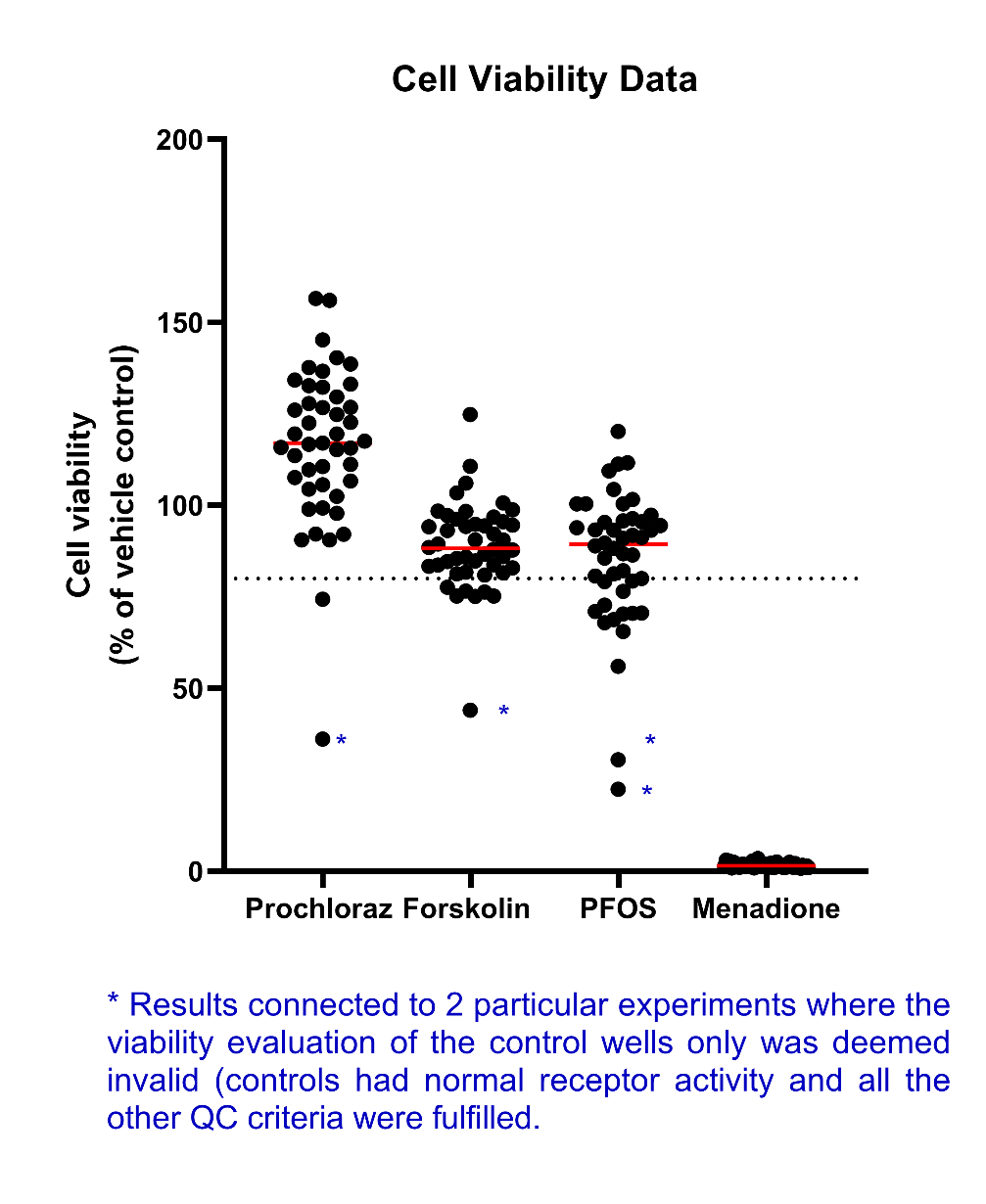


**Figure S6: Correlation analysis between H295R cell viability & observed Androgen activity for PFOS (validation experiments only):** No correlation could be observed between the two variables (R^2^=0.01).
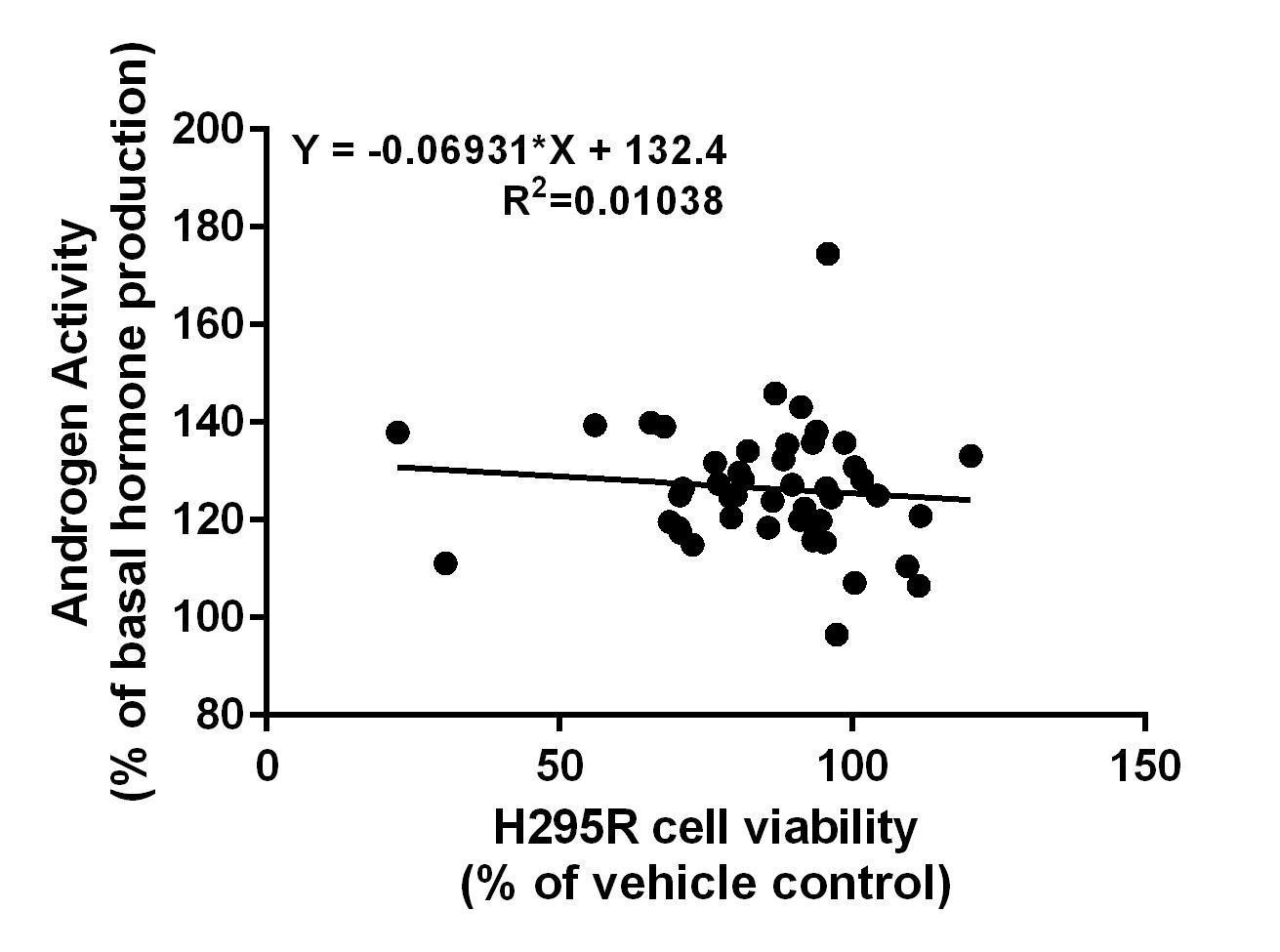


**Figure S7 Additional same-well cell viability measurements for the H295R cells, as well as the ER and AR U2OS CALUX cells for the chemicals shown in Figure 4.** Reported concentrations reflect either the final well-concentration in the H295R assay (H295R cell viability) or the mean final concentration in the CALUX wells (CALUX cell viability). The graphs depict the results of 3 biologically independent experiments made up of 3 technical replicates (n=9 with the exception of letrozole, where n=6 for the following concentrations: 3*10^-12^, 3*10^-11^ & 1*10^-10^ M and aminoglutethimide, where n=6 for 3*10^-4^ M and n=3 for 10^-7^M). Each point represents the mean of means ± the standard error of that mean of means (SEM).


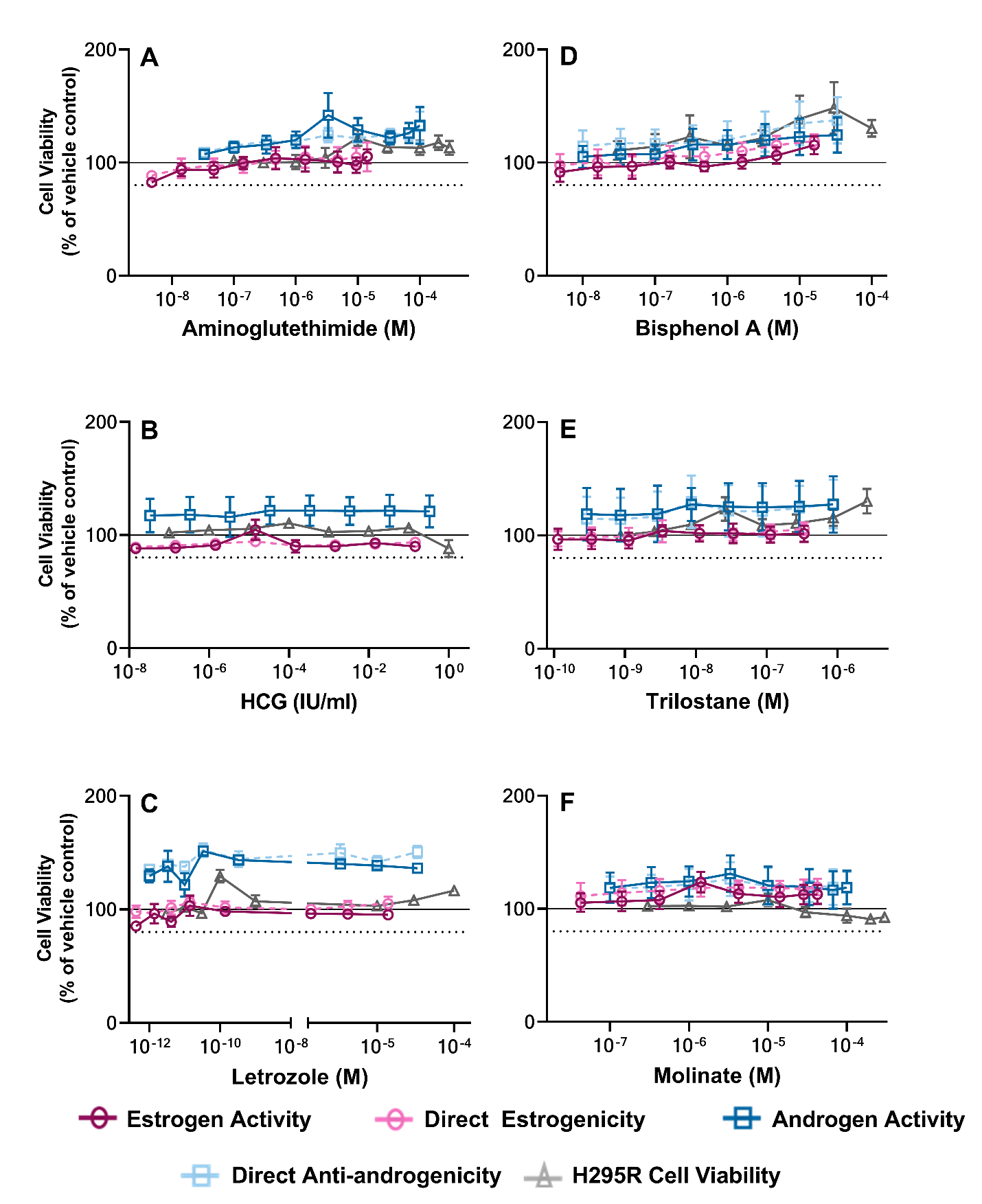


**Figure S8 Androgen activity and direct anti-androgenicity for the 3 top treatment concentrations of molinate (A), letrozole (B) and atrazine (C).** Each curve corresponds to a single biological replicate and each point represents the mean measurement in each experiment ± the standard deviation (SD). Reported concentrations reflect the final well-concentration in the H295R assay, without accounting for the dilution into AR CALUX cells.

**

**

**Table S2: Classification of chemicals in confusion matrices**

| **True positives** | **True negatives** | **False positives** | **False negatives** |
| --- | --- | --- | --- |
| **OECD results as true effect (T) versus H295R-CALUX** | | | |
| Prochloraz  Aminoglutethimide | HCG  Benomyl  Atrazine  Molinate  Butylparaben | Forskolin  Trilostane | Letrozole  BPA |
| **OECD results as true effect (E2) versus H295R-CALUX** | | | |
| Forskolin  Trilostane  Atrazine  Molinate  Prochloraz  Aminoglutethimide  Letrozole | HCG,  Benomyl | - | BPA  Butylparaben |
| **OECD + literature based CALUX results as true effect versus H295R-CALUX** | | | |
| Prochloraz  Aminoglutethimide  Atrazine  Molinate  BPA  Butylparaben  Benomyl  Forskolin  Trilostane | HCG | Letrozole | - |
| **OECD + literature based CALUX results as true effect versus H295R-CALUX** | | | |
| Forskolin  Trilostane  Atrazine  Molinate  Prochloraz  Aminoglutethimide  Letrozole  BPA  Butylparaben | HCG,  Benomyl | - | - |
| ***In vivo* as true results (T) versus H295R-CALUX** | | | |
| Prochloraz  Trilostane | HCG  Benomyl  BPA  Atrazine  Molinate  Butylparaben | Aminoglutethimide | Letrozole |
| ***In vivo* as true results (E2) versus H295R-CALUX** | | | |
| Prochloraz  Aminoglutethimide  Atrazine  Trilostane  Letrozole | HCG  Benomyl  BPA  Butylparaben | Molinate | - |
| **True positives** | **True negatives** | **False positives** | **False negatives** |
| ***In vivo* as true versus *in vitro* OECD (T)** | | | |
| Prochloraz | HCG  Atrazine  Molinate  Benomyl  Butylparaben | Aminoglutethimide  Trilostane  BPA  Letrozole |  |
| ***In vivo* as true versus *in vitro* OECD (E2)** | | | |
| Prochloraz  Aminoglutethimide  Atrazine  Letrozole | HCG  Benomyl | Trilostane  Molinate  BPA  Butylparaben | - |

**R-Script for the performance of the Kruskal Wallis analysis, followed by a series of Mann-Whitney U pairwise comparisons**

library(openxlsx)

library(coin)

#Set the working directory

setwd("C:/ Filepath/Results") #where I made the analysis

#reading the file

dat<-read.xlsx("C:/Filepath/Pro_ButPar_Let_Forsk_atr_tril/Statistics for R trilostane.xlsx", sheet="Statistics for R trilostane")

dat2<-read.xlsx("C:/ Filepath/Statistics for R trilostane.xlsx", sheet="Statistics for R trilostane")

#checking the reading

names(dat)

str(dat) #check if it is well read

names(dat2)

str(dat2) #check if it is well read

#new column "perc" to be sure we want to have

dat$percER<-as.numeric(as.character(dat$"%.basal.induction.ER")) #in case you will not have only "numeric", convert it

dat2$percAR<-as.numeric(as.character(dat$"%.basal.induction.(a)AR")) #in case you will not have only "numeric", convert it

#creation of extra column for the test where everything is compared to Vehicle Control + H295R

dat$testER<-paste(dat$Plate.SubstanceER) #create group on which we want to make the KW and pairwise comparisons

dat$groupER<-paste(dat$ER.substance, dat$ER.concentration, dat$H295R.Cells.Present,sep=":")

dat2$testAR<-paste(dat2$Plate.SubstanceAR) #create group on which we want to make the KW and pairwise comparisons

dat2$groupAR<-paste(dat2$AR.Substance, dat2$AR.concentration, dat2$H295R.Cells.Present,sep=":")

#checks

unique(dat$groupER)

dat$groupER[which(dat$groupER=="DMSO:0:TRUE")]<-"DMSO + H295R"

## Creation of data frames for the results

resER<-data.frame(testIDER = character(),

KWER = numeric(),

ComparisonER = character(),

diffER = numeric(),

Wilcox.exactER = numeric(),

Sidak.BonferroniER = numeric())

#Test ER DMSO + H295R

for(te in unique(dat$testER)){#loop on the test

dat.te<-dat[which(dat$testER == te),]

totest<- unique(dat.te$groupER)[-which(unique(dat.te$groupER)=="DMSO + H295R")]#list of the group we want to test against the control

dat.te$groupER<-as.factor(dat.te$groupER)

kw.te<-pvalue(kruskal_test(percER ~ groupER, data = dat.te))

nbtest <- length(unique(dat.te$groupER))-1#for correction for multiplicity of test, as all of them are maybe not relevant we may discuss if it is not better to make the correction manually in Excel afterwards

for(i in totest){

dat.te1<-dat.te[which(dat.te$groupER %in% c("DMSO + H295R",i)),]

dat.te1$groupER<-factor(dat.te1$groupER,levels=c("DMSO + H295R",i))

a<-wilcox_test(percER ~ groupER, data = dat.te1, #I test the groups against each other in the data: dat.te

distribution = "exact", alternative = "two.sided", #exact distribution and two sided test

conf.int = TRUE) #to be noted that with this test with less than 5 values by group you will never be significant

resa<-cbind(testIDER = te, KWER = kw.te, ComparisonER = paste("DMSO + H295R",i,sep=" vs. "), diffER=confint(a)$estimate,

Wilcox.exactER = pvalue(a), Sidak.BonferroniER = 1-(1-pvalue(a))^nbtest)

resER<-rbind(resER,resa)

}}

warnings() #not possible to compute the confidence interval (not enough observations)

write.table(resER,"resERDMSO+H295R.tril.csv",sep=",",row.names=F) #export the results in the file "resERDMSO+H295R.tril.csv"

#checks

unique(dat2$groupAR)

dat2$groupAR[which(dat2$groupAR=="DMSO:0:TRUE")]<-"DMSO + H295R"

## Creation of data frames for the results

resAR<-data.frame(testIDAR = character(),

KWAR = numeric(),

ComparisonAR = character(),

diffAR = numeric(),

Wilcox.exactAR = numeric(),

Sidak.BonferroniAR = numeric())

#Test AR DMSO + H295R

for(te2 in unique(dat2$testAR)){#loop on the test

dat2.te2<-dat2[which(dat2$testAR == te2),]

totest2<- unique(dat2.te2$groupAR)[-which(unique(dat2.te2$groupAR)=="DMSO + H295R")]#list of the group we want to test against the control

dat2.te2$groupAR<-as.factor(dat2.te2$groupAR)

kw2.te2<-pvalue(kruskal_test(percAR ~ groupAR, data = dat2.te2))

nbtest2 <- length(unique(dat2.te2$groupAR))-1 #for correction for multiplicity of test, as all of them are maybe not relevant we may discuss if it is not better to make the correction manually in Excel afterwards

for(j in totest2){

dat2.te1<-dat2.te2[which(dat2.te2$groupAR %in% c("DMSO + H295R",j)),]

dat2.te1$groupAR<-factor(dat2.te1$groupAR,levels=c("DMSO + H295R",j))

b<-wilcox_test(percAR ~ groupAR, data = dat2.te1, #I test the groups against each other in the data: dat2.te

distribution = "exact", alternative = "two.sided", #exact distribution and two sided test

conf.int = TRUE) #to be noted that with this test with less than 5 values by group you will never be significant

resb<-cbind(testIDAR = te2, KWAR = kw2.te2, ComparisonAR = paste("DMSO + H295R",j,sep=" vs. "), diffAR=confint(b)$estimate,

Wilcox.exactAR = pvalue(b), Sidak.BonferroniAR = 1-(1-pvalue(b))^nbtest2)

resAR<-rbind(resAR,resb)

}}

warnings() #not possible to compute the confidence interval (not enough observations)

write.table(resAR,"resARDMSO+H295R.tril.csv",sep=",",row.names=F) #export the results in the file "resARDMSO+H295R.tril.csv"

setwd("C:/filepath/Results") #where I made the analysis

#reading the file

dat<-read.xlsx("C:/filepath/Statistics for R trilostane.xlsx", sheet="Statistics for R trilostane")

dat2<-read.xlsx("C:/filepath/Statistics for R trilostane.xlsx", sheet="Statistics for R trilostane")

#checking the reading

names(dat)

str(dat) #check if it is well read

names(dat2)

str(dat2) #check if it is well read

#new column "perc" to be sure we want to have

dat$percER<-as.numeric(as.character(dat$"%.basal.induction.ER")) #in case you will not have only "numeric", convert it

dat2$percAR<-as.numeric(as.character(dat$"%.basal.induction.(a)AR")) #in case you will not have only "numeric", convert it

#creation of extra column for the test where everything is compared to Vehicle Control + H295R

dat$testER<-paste(dat$Plate.SubstanceER, dat$H295R.Cells.Present, sep="-") #create group on which we want to make the KW and pairwise comparisons

dat$groupER<-paste(dat$ER.substance, dat$ER.concentration, sep=":")

dat2$testAR<-paste(dat2$Plate.SubstanceAR, dat$H295R.Cells.Present, sep="-") #create group on which we want to make the KW and pairwise comparisons

dat2$groupAR<-paste(dat2$AR.Substance, dat2$AR.concentration, sep=":")

#checks

unique(dat$groupER)

dat$groupER[which(dat$groupER=="DMSO:0")]<-"DMSO"

unique(dat2$groupAR)

dat2$groupAR[which(dat2$groupAR=="DMSO:0")]<-"DMSO"

## Creation of data frames for the results

resER<-data.frame(testIDER = character(),

KWER = numeric(),

ComparisonER = character(),

diffER = numeric(),

Wilcox.exactER = numeric(),

Sidak.BonferroniER = numeric())

resAR<-data.frame(testIDAR = character(),

KWAR = numeric(),

ComparisonAR = character(),

diffAR = numeric(),

Wilcox.exactAR = numeric(),

Sidak.BonferroniAR = numeric())

#Test ER DMSO

for(te in unique(dat$testER)){#loop on the test

dat.te<-dat[which(dat$testER == te),]

totest<- unique(dat.te$groupER)[-which(unique(dat.te$groupER)=="DMSO")]#list of the group we want to test against the control

dat.te$groupER<-as.factor(dat.te$groupER)

kw.te<-pvalue(kruskal_test(percER ~ groupER, data = dat.te))

nbtest <- length(unique(dat.te$groupER))-1#for correction for multiplicity of test, as all of them are maybe not relevant we may discuss if it is not better to make the correction manually in Excel afterwards

for(i in totest){

dat.te1<-dat.te[which(dat.te$groupER %in% c("DMSO",i)),]

dat.te1$groupER<-factor(dat.te1$groupER,levels=c("DMSO",i))

a<-wilcox_test(percER ~ groupER, data = dat.te1, #I test the groups against each other in the data: dat.te

distribution = "exact", alternative = "two.sided", #exact distribution and two sided test

conf.int = TRUE) #to be noted that with this test with less than 5 values by group you will never be significant

resa<-cbind(testIDER = te, KWER = kw.te, ComparisonER = paste("DMSO",i,sep=" vs. "), diffER=confint(a)$estimate,

Wilcox.exactER = pvalue(a), Sidak.BonferroniER = 1-(1-pvalue(a))^nbtest)

resER<-rbind(resER,resa)

}}

warnings() #not possible to compute the confidence interval (not enough observations)

write.table(resER,"resERDMSO.tril.csv",sep=",",row.names=F) #export the results in the file "resERDMSO.tril.csv"

#Test AR DMSO

for(te2 in unique(dat2$testAR)){#loop on the test

dat2.te2<-dat2[which(dat2$testAR == te2),]

totest2<- unique(dat2.te2$groupAR)[-which(unique(dat2.te2$groupAR)=="DMSO")]#list of the group we want to test against the control

dat2.te2$groupAR<-as.factor(dat2.te2$groupAR)

kw2.te2<-pvalue(kruskal_test(percAR ~ groupAR, data = dat2.te2))

nbtest2 <- length(unique(dat2.te2$groupAR))-1 #for correction for multiplicity of test, as all of them are maybe not relevant we may discuss if it is not better to make the correction manually in Excel afterwards

for(j in totest2){

dat2.te1<-dat2.te2[which(dat2.te2$groupAR %in% c("DMSO",j)),]

dat2.te1$groupAR<-factor(dat2.te1$groupAR,levels=c("DMSO",j))

b<-wilcox_test(percAR ~ groupAR, data = dat2.te1, #I test the groups against each other in the data: dat2.te

distribution = "exact", alternative = "two.sided", #exact distribution and two sided test

conf.int = TRUE) #to be noted that with this test with less than 5 values by group you will never be significant

resb<-cbind(testIDAR = te2, KWAR = kw2.te2, ComparisonAR = paste("DMSO",j,sep=" vs. "), diffAR=confint(b)$estimate,

Wilcox.exactAR = pvalue(b), Sidak.BonferroniAR = 1-(1-pvalue(b))^nbtest2)

resAR<-rbind(resAR,resb)

}}

warnings() #not possible to compute the confidence interval (not enough observations)

write.table(resAR,"resARDMSO.tril.csv",sep=",",row.names=F) #export the results in the file "resARDMSO.tril.csv"
